# Serial scanning electron microscopy of anti-PKHD1L1 immuno-gold labeled mouse hair cell stereocilia bundles

**DOI:** 10.1038/s41597-020-0509-4

**Published:** 2020-06-17

**Authors:** Maryna V. Ivanchenko, Marcelo Cicconet, Hoor Al Jandal, Xudong Wu, David P. Corey, Artur A. Indzhykulian

**Affiliations:** 1 Department of Neurobiology, Harvard Medical School, 220 Longwood Avenue, Boston, MA 02115 USA; 2 Image and Data Analysis Core, Harvard Medical School, 43 Shattuck St, Boston, MA 02115 USA; 3 Northeastern University, 360 Huntington Avenue, Boston, MA 02115 USA; 4 Department of Otolaryngology, Harvard Medical School and Massachusetts Eye and Ear, 243 Charles St, Boston, MA 02114 USA

**Keywords:** Immunohistochemistry, 3-D reconstruction, Hair cell, Scanning electron microscopy

## Abstract

Serial electron microscopy techniques have proven to be a powerful tool in biology. Unfortunately, the data sets they generate lack robust and accurate automated segmentation algorithms. In this data descriptor publication, we introduce a serial focused ion beam scanning electron microscopy (FIB-SEM) dataset consisting of six outer hair cell (OHC) stereocilia bundles, and the supranuclear part of the hair cell bodies. Also presented are the manual segmentations of stereocilia bundles and the gold bead labeling of PKHD1L1, a coat protein of hair cell stereocilia important for hearing in mice. This depository includes all original data and several intermediate steps of the manual analysis, as well as the *MATLAB* algorithm used to generate a three-dimensional distribution map of gold labels. They serve as a reference dataset, and they enable reproduction of our analysis, evaluation and improvement of current methods of protein localization, and training of algorithms for accurate automated segmentation.

## Abstract (summary)

**Table:** 

Measurement(s)	outer hair cell • cochlear hair cell
Technology Type(s)	scanning electron micrograph
Factor Type(s)	anti-PKHD1L1 labeling distribution
Sample Characteristic - Organism	Mus musculus

Machine-accessible metadata file describing the reported data: 10.6084/m9.figshare.12210158

## Background & Summary

Our senses of hearing and balance are secured by the sensory cells of the inner ear, the hair cells, which transform the mechanical stimuli of sound or head movement into electrical signals our brain can understand through a process called mechanoelectrical transduction (MET). Each hair cell carries a bundle of ~100 highly specialized, actin-filled microvillus-like projections called stereocilia. The heights of stereocilia are precisely organized within the bundle from short to tall; they pivot about their insertion point into the apical end of the hair cell upon mechanical stimulation. Stereocilia are cross-linked by a number of transient or permanent links, collectively termed as stereociliary ‘surface specializations’, which are identified with electron microscopy and ensure bundle integrity^1^. A list of proteins were shown to be important for the hair cell stereocilia bundle structure and function, many of which were localized to the bundle using variations of immunogold labeling protocols, and observed with electron microscopy (EM) techniques, mostly using transmission electron microscopy (TEM)^2–15^ and more lately using scanning electron microscopy (SEM)^16–21^. In the most recent years though, serial electron microscopy techniques have become more accessible for use in biology, mounting a new body of work using 3D EM approaches^17,22,23^.

The hair cell stereocilia bundle is a polarized structure detecting bundle deflections in a certain direction, commonly known as the MET axis^10,16,24^. The MET function is mediated by tip links, which are tiny filaments connecting the side of the taller stereocilium to the tip of the shorter one^10,12,16,18^. Upon bundle deflection, tip links force the MET channel at the tips of stereocilia to open. As such, the MET axis for any given pair of stereocilia can vary depending on the orientation of the stereocilia and the tip links connecting them. This becomes more obvious in outer hair cell bundles, which have a more pronounced “V-shape” stereocilia arrangement within the bundle^17^.

During development, early postnatal hair cells have their primary cilium, a kinocilium, which later degenerates upon maturation of the hair bundle. Developing stereocilia emerge at the apical end of the hair cell resembling a very similar appearance to microvilli, but over time a subset of these microvilli closest to the kinocilium transform into stereocilia: they elongate, thicken, and their actin filaments get densely packed, while the rest of the microvilli further away from the kinocilium are resorbed over time^25^. Upon completion of this process just three rows of stereocilia are left at the apical surface of the hair cell, as seen in adult mouse hair cells. The functional significance of the shorter stereocilia (or microvilli) past the third row in developing bundles is unknown. While Ca^2+^-imaging experiments conducted on rat and mouse hair cell stereocilia bundles show MET activity in the second and third rows of stereocilia^24,26^, no convincing evidence currently exists suggesting presence of MET activity in shorter rows of stereocilia (and/or microvilli) past the third row in developing stereocilia bundles. Because of their transient nature, these structures are often considered of no major significance to the function of adult stereocilia bundles.

In a recent study, we showed that one type of stereocilia surface specialization, the stereociliary coat, is formed at least in part by a newly identified stereociliary protein, Polycystic Kidney and Hepatic Disease 1-Like 1 (PKHD1L1)^17^. This study was carried out using serial scanning electron microscopy of hair cell bundles immunolabeled with anti-PKHD1L1 and gold bead conjugated antibodies. We showed that PKHD1L1 is located at the tips of stereocilia, and quantified the gold bead distribution in different regions of the surfaces of stereocilia. PKHD1L1-deficient mice lack the surface coat at the upper but not lower regions of stereocilia, confirming that PKHD1L1 is a component of the surface coat, and they develop progressive hearing loss, showing that PKHD1L1 is required for normal hearing in mice.

**Fig. 1:**
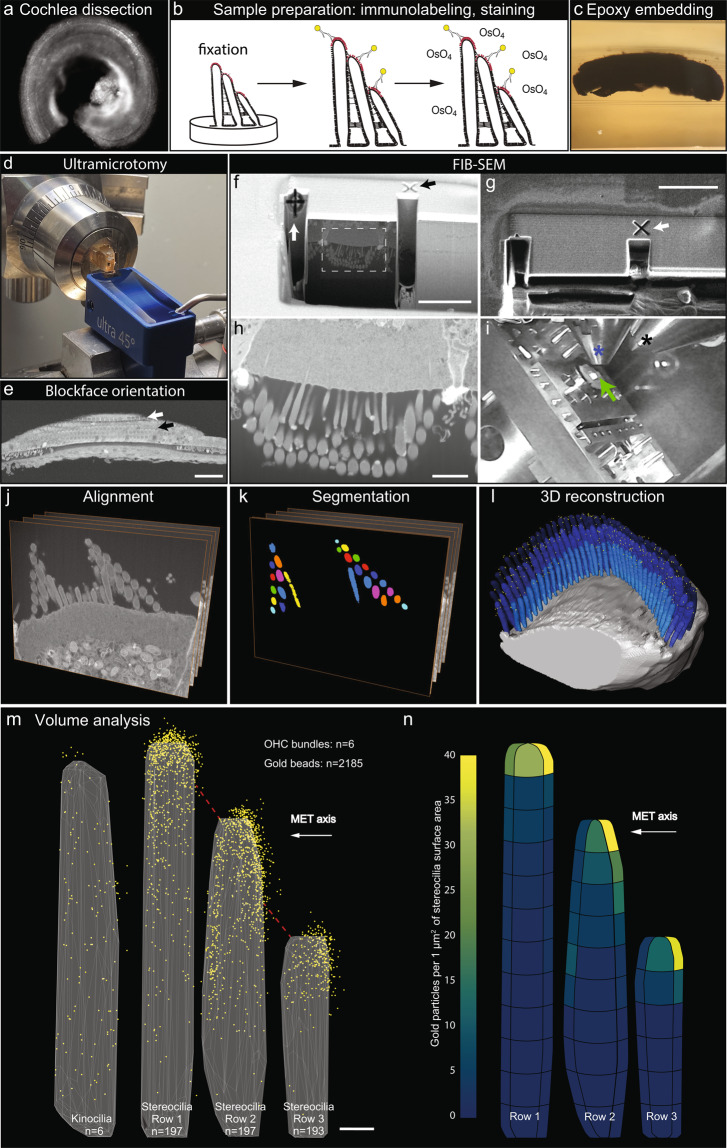
Illustration of the workflow outlining the major steps of the current study. (**a)** Organ of Corti dissection, tissue fixation. (**b**) Immunogold labeling and EM staining with heavy metals. Yellow circles were added to schematically represent gold beads. (**c**) Epoxy resin embedding. (**d**) Tissue block trimming and sectioning to approach the hair cell stereocilia bundles using ultramicrotomy. (**e)** Following transmission electron microscopy imaging of ultrathin sections to confirm proper tissue orientation and location within the resin block, the block was mounted for FIB-SEM, and observed with an insertable backscatter detector to identify hair cell bodies. *White arrow*, IHC region; *black arrow*, OHC region. The hair cell bodies have been partially sectioned away, with their stereocilia bundles still embedded within the resin block and pointing down, as shown in *f*. (**f–i**) FIB-SEM imaging procedure. The volume of interest was first prepared for serial imaging by placing fiducial markers and milling trenches to approach the cell, as shown in *f-g*. Next, the area of interest was imaged at higher magnification as shown in *h*. *Black arrow* in *f* points to the same fiducial marker, as the *white arrow* in *g*, used to align the ion beam milling process. *White arrow* in *f* points to the fiducial marker used to align the SEM serial imaging area outlined by white dashed lines, also shown in *h*. Panel *i* shows the microscope chamber with the sample (*green arrow*) mounted on the microscope stage, tilted to 52°, and brought close to the FIB source (*black asterisk*) and the SEM source (*blue asterisk*). Images *f, h* were acquired by the SEM beam and are at 52° tilt from the image in panel *g*, acquired by the ion beam. (**j**–**l**), Image processing steps: image alignment (*j*), stereocilia and gold bead segmentation (*k*), and 3D reconstruction (*l*) using Dragonfly and Amira software packages. (**m,n**), Volume analysis using a custom *MATLAB* algorithm allowed to generate gold bead distribution maps (*m*) and quantify the gold beads per stereocilia surface area (*n*). Panels *m-n* are published with permission from the original study^17^. Scale bars: *e*, 50 μm; *f-g*, 5 μm; *h*, 2 μm; m, 200 nm.

To quantitatively evaluate the distribution of PKHD1L1 and its labeling density at the surface of stereocilia, we used FIB-SEM to collect serial electron microscopy data sets of stereocilia bundles from postnatal day 4 (P4) outer hair cells (OHCs). FIB-SEM uses a focused gallium-ion beam to etch the surface in defined steps of just a few nanometers thick, and SEM with backscatter detection to image the heavy metal counterstain and 10-nm gold beads at each freshly etched surface. Using this sequential milling and imaging approach, we generated a number of serial EM data sets, which were then segmented and three dimensionally reconstructed using Dragonfly and Amira software packages. Although a number of organ of Corti samples were carefully evaluated using TEM, only one sample was chosen for further FIB-SEM imaging. The specificity of anti-PKHD1L1 labeling has been evaluated as part of the original study using light microscopy, SEM and TEM^17^. The serial EM datasets in this submission are of six closely located OHCs, collected from the middle cochlear turn of a P4 mouse cochlea. An overview of the sample preparation, data collection, and analysis steps are provided in Fig. 1. In addition, movies illustrating 3D localization of PKHD1L1 in immunogold labeled outer hair cell stereocilia bundles using FIB‐ SEM (postnatal day 4) in a single OHC, and 3D cumulative distribution map of anti‐PKHD1L1 immuno‐gold beads from six mouse OHC stereocilia bundles (postnatal day 4) were deposited at figshare^27^. Both movies are published with permission from the original study^17^.

In our datasets, the pixel size varied between 1.95 and 2.75 nm, and the milling step varied between 10 and 20 nm; see Table 1 for details. The manual segmentation workflow implemented in this study produced stacks containing the kinocilium, three rows of stereocilia (manually assigned to the tallest, middle and short rows), and the gold beads.

**Table 1:** Summary of imaging data parameters.

Parameters	Cell #1^28^	Cell #2^36^	Cell #3^44^	Cell #4^51–53^		Cell #5^60^	Cell #6^28^
				Part 1^51^	Part 2^52^		
Pixel size, X (nm) Y (nm) Z step (nm)	2.43	1.95	2.60	1.95	1.95	2.75	2.43
	2.43	1.95	2.60	1.95	1.95	2.75	2.43
	15	10	15	15	15	20	15
# of slices (images)	388	311	208	224	144	233	388
Acquisition HFW (µm)	14.9	7.98	7.99	7.98	7.97	8.46	14.9
**Image aspect ratio:**							
Width (pixels)	6144	4096	3072	4096	4096	3072	6144
Height (pixels)	4096	3536	2048	3536	3536	2048	4096

All datasets referenced in this study were deposited at *The Cell Image Library* repository^28–75^, and can be accessed using the links provided in Table 2. This submission is accompanied by 48 datasets, six of which are the original FIB-SEM datasets of six stereocilia bundles^28,36,44,51,52,60^, one test dataset used to validate the performance of the *MATLAB* algorithm^72–74^, and one dataset deposition of the *MATLAB* scripts used to perform the analysis^75^. All remaining dataset depositions are the files of various intermediate steps of data analysis and segmentation performed in this study. The files are grouped into eight dataset groups, each accessible via a dedicated group link provided in Table 2, as well as via individual links. Dataset groups 1–6 consist of files related to cells 1–6 respectively, dataset group 7 consists of files related to the test cell, and dataset 8 consists of files composing the *MATLAB* script.

**Table 2:** Summary of datasets accompanying this study.

**Dataset group 1** (consisting of 8 files).	Group link: http://cellimagelibrary.org/groups/50680.	
1.1_Cell_1_and_Cell_6_Raw_Image_Stack^28^	http://cellimagelibrary.org/images/50680;	10.7295/W9CIL50680
1.2_Cell_1_Stack_Aligned^29^	http://cellimagelibrary.org/images/50681;	10.7295/W9CIL50681
1.3_Cell_1_Stack_Cropped^30^	http://cellimagelibrary.org/images/50682;	10.7295/W9CIL50682
1.4_Cell_1_Stack_Filtered^31^	http://cellimagelibrary.org/images/50683;	10.7295/W9CIL50683
1.5_Cell_1_Stereocilia_Segmentation^32^	http://cellimagelibrary.org/images/50684;	10.7295/W9CIL50684
1.6_Cell_1_GoldParticle_Segmentation^33^	http://cellimagelibrary.org/images/50685;	10.7295/W9CIL50685
1.7_Cell_1_Surface_Rendering^34^	http://cellimagelibrary.org/images/50686;	10.7295/W9CIL50686
1.8_Cell_1_MATLAB_Volume_Analysis_Results^35^	http://cellimagelibrary.org/images/50687;	10.7295/W9CIL50687
**Dataset group 2** (consisting of 8 files).	Group link: http://cellimagelibrary.org/groups/50688.	
2.1_Cell_2_Raw_Image_Stack^36^	http://cellimagelibrary.org/images/50688;	10.7295/W9CIL50688
2.2_Cell_2_Stack_Cropped^37^	http://cellimagelibrary.org/images/50689;	10.7295/W9CIL50689
2.3_Cell_2_Stack_Aligned^38^	http://cellimagelibrary.org/images/50690;	10.7295/W9CIL50690
2.4_Cell_2_Stack_Filtered^39^	http://cellimagelibrary.org/images/50691;	10.7295/W9CIL50691
2.5_Cell_2_Stereocilia_Segmentation^40^	http://cellimagelibrary.org/images/50692;	10.7295/W9CIL50692
2.6_Cell_2_GoldParticle_Segmentation^41^	http://cellimagelibrary.org/images/50693;	10.7295/W9CIL50693
2.7_Cell_2_Surface_Rendering^42^	http://cellimagelibrary.org/images/50695;	10.7295/W9CIL50695
2.8_Cell_2_MATLAB_Volume_Analysis_Results^43^	http://cellimagelibrary.org/images/50696;	10.7295/W9CIL50696
**Dataset group 3** (consisting of 7 files).	Group link: http://cellimagelibrary.org/groups/50697.	
3.1_Cell_3_Raw_Image_Stack^44^	http://cellimagelibrary.org/images/50697;	10.7295/W9CIL50697
3.2_Cell_3_Stack_Cropped_Aligned^45^	http://cellimagelibrary.org/images/50698;	10.7295/W9CIL50698
3.3_Cell_3_Stack_Filtered^46^	http://cellimagelibrary.org/images/50699;	10.7295/W9CIL50699
3.4_Cell_3_Stereocilia_Segmentation^47^	http://cellimagelibrary.org/images/50700;	10.7295/W9CIL50700
3.5_Cell_3_GoldParticle_Segmentation^48^	http://cellimagelibrary.org/images/50701;	10.7295/W9CIL50701
3.6_Cell_3_Surface_Rendering^49^	http://cellimagelibrary.org/images/50702;	10.7295/W9CIL50702
3.7_Cell_3_MATLAB_Volume_Analysis_Results^50^	http://cellimagelibrary.org/images/50703;	10.7295/W9CIL50703
**Dataset group 4** (consisting of 9 files).	Group link: http://cellimagelibrary.org/groups/50704.	
4.1_Cell_4_Raw_Image_Stack_Part1^51^	http://cellimagelibrary.org/images/50704;	10.7295/W9CIL50704
4.2_Cell_4_Raw_Image_Stack_Part2^52^	http://cellimagelibrary.org/images/50705;	10.7295/W9CIL50705
4.3_Cell_4_Raw_Image_Stack^53^	http://cellimagelibrary.org/images/50706;	10.7295/W9CIL50706
4.4_Cell_4_Stack_Cropped_Aligned^54^	http://cellimagelibrary.org/images/50707;	10.7295/W9CIL50707
4.5_Cell_4_Stack_Filtered^55^	http://cellimagelibrary.org/images/50708;	10.7295/W9CIL50708
4.6_Cell_4_Stereocilia_Segmentation^56^	http://cellimagelibrary.org/images/50709;	10.7295/W9CIL50709
4.7_Cell_4_GoldParticle_Segmentation^57^	http://cellimagelibrary.org/images/50710;	10.7295/W9CIL50710
4.8_Cell_4_Surface_Rendering^58^	http://cellimagelibrary.org/images/50711;	10.7295/W9CIL50711
4.9_Cell_4_MATLAB_Volume_Analysis_Results^59^	http://cellimagelibrary.org/images/50712;	10.7295/W9CIL50712
**Dataset group 5** (consisting of 7 files). Group link:	Group link: http://cellimagelibrary.org/groups/50713.	
5.1_Cell_5_Raw_Image_Stack^60^	http://cellimagelibrary.org/images/50713;	10.7295/W9CIL50713
5.2_Cell_5_Stack_Cropped_Aligned^61^	http://cellimagelibrary.org/images/50714;	10.7295/W9CIL50714
5.3_Cell_5_Stack_Filtered^63^	http://cellimagelibrary.org/images/50715;	10.7295/W9CIL50715
5.4_Cell_5_Stereocilia_Segmentation^63^	http://cellimagelibrary.org/images/50716;	10.7295/W9CIL50716
5.5_Cell_5_GoldParticle_Segmentation^64^	http://cellimagelibrary.org/images/50717;	10.7295/W9CIL50717
5.6_Cell_5_Surface_Rendering^65^	http://cellimagelibrary.org/images/50718;	10.7295/W9CIL50718
5.7_Cell_5_MATLAB_Volume_Analysis_Results^66^	http://cellimagelibrary.org/images/50719;	10.7295/W9CIL50719
**Dataset group 6** (consisting of 6 files).	Group link: http://cellimagelibrary.org/groups/50720.	
1.1_Cell_1_and_Cell6_Raw_Image_Stack^28^	http://cellimagelibrary.org/images/50680;	10.7295/W9CIL50680
6.2_Cell_6_Stack_Aligned_Cropped^67^	http://cellimagelibrary.org/images/50720;	10.7295/W9CIL50720
6.3_Cell_6_Stereocilia_Segmentation^68^	http://cellimagelibrary.org/images/50721;	10.7295/W9CIL50721
6.4_Cell_6_GoldParticle_Segmentation^69^	http://cellimagelibrary.org/images/50722;	10.7295/W9CIL50722
6.5_Cell_6_Surface_Rendering^70^	http://cellimagelibrary.org/images/50723;	10.7295/W9CIL50723
6.6_Cell_6_MATLAB_Volume_Analysis_Results^71^	http://cellimagelibrary.org/images/50724;	10.7295/W9CIL50724
**Dataset group 7** (consisting of 3 files).	Group link: http://cellimagelibrary.org/groups/50725.	
7.1_Test_Cell_Stereocilia_Segmentation^72^	http://cellimagelibrary.org/images/50725;	10.7295/W9CIL50725
7.2_Test_Cell_GoldParticle_Segmentation^73^	http://cellimagelibrary.org/images/50726;	10.7295/W9CIL50726
7.3_Test_Cell_MATLAB_Volume_Analysis_Results^74^	http://cellimagelibrary.org/images/50727;	10.7295/W9CIL50727
**Dataset 8**. *MATLAB* script.		
Dataset8_MATLAB script^75^	http://cellimagelibrary.org/images/50728;	10.7295/W9CIL50728

Because the terms *‘stereocilia’* and *‘kinocilia’* are used very often throughout the manuscript, and some steps of our analysis are common for both structures, we implemented a terminology as outlined below. When referring to the hair cell primary cilia only, we call them ‘*kinocilia’*, as commonly known in the field. When referring to stereocilia only, we call them stereocilia. When referring to kinocilia and stereocilia together (while describing the segmentation, and other steps of the analysis that don’t distinguish between the two structures) we refer to them jointly as ‘*cilia’*. While describing the details of the *MATLAB* script, designed to work with any tower-shaped object (cilia or not), we refer to these structures as ‘*towers’*.

## Methods

This section contains a more detailed description of the steps and procedures used to produce the results previously reported as an original study^17^. All experiments were carried out in compliance with ethical regulations and approved by the Animal Care Committees of the Harvard Medical School and Massachusetts Eye and Ear.

### Cochlear dissection, sample preparation, epoxy embedding, FIB-SEM image acquisition

Organ of Corti explants were dissected at P4 in L-15 medium (GIBCO), fixed with 4% formaldehyde in HBSS for 2 hours at room temperature and washed in HBSS (Fig. 1a). After the fixation, tectorial membrane overlaying the hair cells was pulled away to expose the sensory epithelium. Cochleas were immunolabeled as previously described^17^. Briefly, after nonspecific binding sites were blocked by 10% goat serum in Ca^2+^, Mg^2+^-free HBSS for 2 h at room temperature, samples were incubated at 4 °C with primary antibodies diluted in blocking solution 1:200 (rabbit anti-PKHD1L1 antibody, NovusBio #NBP2-13765) in a wet chamber overnight, followed by several rinses in Ca^2+^, Mg^2+^-free HBSS. Next, samples were incubated in blocking solution for 30 min at room temperature, and overnight at 4 °C with secondary antibody solution (gold Conjugate EM Goat F(ab′)2 anti-rabbit IgG: 10 nm gold (BB International # 14216), diluted 1:30 in blocking solution).

Following the secondary antibody application samples were rinsed in Ca^2+^, Mg^2+^-free HBSS (3×), postfixed with 2.5% glutaraldehyde in 0.1 M cacodylate buffer (pH 7.2) supplemented with 2 mM CaCl_2_ containing 1% tannic acid for 1–2 hours at room temperature. Following a triple rinse in cacodylate buffer, they were postfixed and stained with 1% osmium tetroxide/1.5% potassium ferrocyanide in 0.1 M cacodylate buffer for 2 hours at room temperature in the dark. Explants were then washed three times in 0.1-M cacodylate buffer (pH 7.2), followed by a brief wash in distilled water. They were then dehydrated in an ascending series of ethanol solutions (50%, 70%, 95%, 100%), twice for each concentration, for 10 min each step. Samples were then equilibrated in propylene oxide 3 times for 15 min. Next, they were infiltrated and embedded in propylene oxide/epoxy resin mixtures (Araldite 502/Embed-812 embedding media, EMS) as follows: resin and propylene oxide (1:3) for 4–6 h; followed by resin and propylene oxide (1:1) for 4–8 h; then resin and propylene oxide (3:1) for 8 h. Tissue pieces were then positioned in molds (EMS, 70900) for 24 h and polymerized in the oven at 60 °C for 48 h (for more details on reagents and solutions, see Table 3).

**Table 3:** Reagents and solutions used for sample preparation.

	Reagent	Final solution
Buffers	Leibovitz’s L-15 medium, no phenol red (GIBCO, 21083-027)	
	Hank’s Balanced Salt Solution, with calcium, magnesium, no phenol red, 500 ml (GIBCO, 14025092)	
	Hank’s Balanced Salt Solution, no calcium, no magnesium, no phenol red, 500 ml (GIBCO, 14175095)	
	Sodium Cacodylate Buffer, 0.2 M, pH 7.4, 500 ml (EMS, 11652)	Sodium Cacodylate Buffer, 0.1 M, pH 7.4
Fixatives	16% formaldehyde, 10 ml (EMS 15700)	4% formaldehyde, in HBSS with calcium, magnesium, no phenol red (pH 7.2)
	2.5% Glutaraldehyde in 0.1 M Sodium Cacodylate Buffer, pH 7.4, 10 ml (EMS, 15960)	2.5% glutaraldehyde in 0.1 M cacodylate buffer (pH 7.2) containing 1% tannic acid and supplemented with 2 mM CaCl_2_
	Tannic Acid, Reagent, A.C.S., 100 g (EMS, 21710)	
Blocking solution	Goat serum, 10 g (Jackson Immunoresearch, 005-000-121)	10% goat serum in HBSS, no calcium, no magnesium, no phenol red (pH 7.2)
Antibodies	Rabbit anti-PKHD1L1 antibody, (NovusBio, NBP2-13765)	1:200 dilution in 10% goat serum blocking solution
	Gold Conjugate EM Goat F(ab′)2 anti-rabbit IgG:10 nm gold (BB International #14216)	1:30 dilution in 10% goat serum blocking solution
Staining solutions	Potassium Ferrocyanide Trihydrate, 50 g (EMS, 26604-01)	1% osmium tetroxide/1.5% potassium ferrocyanide in 0.1 M cacodylate buffer, (pH 7.2)
	4% aqueous osmium tetroxide, 5 ml (EMS, 26604-01)	
Dehydration and embedding solutions	Ethanol, 200 proof.	30%, 50%, 80%, 100% ethanol in distilled water
	Propylene oxide, 450 ml (EMS, 20401)	100% Propylene Oxide Araldite-EMbed + propylene oxide (1:3) Araldite-EMbed + propylene oxide (1:1) Araldite-EMbed + propylene oxide (3:1)
	Araldite-EMbed (Mollenhauer’s Kit): EMbed-812, 25 ml, Araldite 502. 15 ml, DDSA; 55 ml DMP-30, 1.5–1.9 ml (EMS, 13940)	

The embedded specimen was exposed using a Reichert Ultracut S ultramicrotome equipped with diamond cutting and diamond trimming knives (Fig. 1c,d). Upon approaching the region of interest, the sample orientation exposing the rows of inner or outer hair cell stereocilia bundles was confirmed by viewing methylene blue-stained semithin sections on a light microscope, and by imaging ultrathin sections on TEM when necessary.

Blocks with the embedded tissue were then cut with a razor blade or a coping saw to a smaller size (between 1–5 mm tall) reducing their height upon mounting. Special care was given to protect the working surface with exposed hair cells and stereocilia bundles from the dust generated by cutting the block, typically by wrapping the block with a wide piece of parafilm, also ensuring the film does not contact the working surface either. The cut resin block was then mounted on aluminum stubs using conductive carbon adhesive tabs, with the working surface facing upwards. All surfaces of the resin block, except for the working surface, were carefully covered with colloidal silver (or carbon) paint to increase sample conductivity, minimizing its charging, then sputter-coated with at least 5 nm of platinum.

Samples were observed on a FEI Helios 660 FIB SEM microscope with an insertable CBS backscatter detector to identify the tissue within the block as seen on Fig. 1e, and locate the region of interest. The sample was brought to the eucentric point (where the electron beam and the ion beam coincide) at ~4 mm working distance to allow for the ion beam image (typically operated at 30 kV and 80 pA) to coincide with the electron beam image. Next, a thin layer of platinum or tungsten was locally deposited on the desired milling area (typically ~10 × 30 μm^2^) using the electron beam (at ~13 nA) to protect the sample. Although inefficient, the SEM metal deposition was always used to deposit a thin protective layer of platinum before switching to ion beam deposition. Next, a more efficient, but more damaging ion beam (~0.8 nA) deposition procedure was carried out, aiming for a final deposition of ~1 μm of platinum. The deposited platinum is clearly visible as the overlaying thick white stripe of protective metal coat on Fig. 1f,g. A very similar procedure was used to prepare a small nearby area where the two fiducial markers were etched (arrows on Fig. 1f,g). The trenches exposing the cell-substrate interface in the cross-section were milled using the focused ion beam set to 30 kV and 2.5 nA.

After preparing the volume for sectioning, serial images of the block face were acquired by repeated cycles of sample milling and imaging using the Auto Slice & View G3 operating software (FEI) with a milling step set between 10–20 nm. During this process, the ion beam was set to 30 kV, 80 pA, and 1 μs dwell time. The electron beam images were usually acquired in an immersion mode by detecting backscattered electrons using the BSE-TLD detector, with an electron beam set to 3 kV at 1.6 nA and 5–10 μs dwell time.

A total of six OHCs were imaged with FIB-SEM, and were used to create this dataset. Seven stacks of ~150–400 slices at the pixel size of 1.95–2.75 nm and 10–20 nm z-step size were collected using the Auto Slice and View function of the FEI acquisition software (see Table 1 for details outlining some of the acquisition parameters).

### Image processing

Collected 8-bit serial TIFF images were combined in *ImageJ* software into a stack, then cropped to reduce the file size below 4 Gb in order to improve the performance of the 3D segmentation and rendering software. Figure 1j–n summarizes the steps of the data analysis described in detail in the sections below. All procedures were carried out on Dell T5810 workstation equipped with an 8-Core 3.4 GHz Intel Xeon E5-1680 v4 Processor, NVIDIA Quadro M5000 8 Gb graphics card, 64 Gb RAM and solid-state drives.

In one case (see Cell #4^51,52^, Table 1) the entire volume was collected through two consecutive Slice and View sessions, later combined for the analysis^53^. The voxel size was noted from the Slice and View software upon completion of the project, also confirmed from the *‘Projectlog.log’* file, in which ‘*targetthickness’* defines the milling (Z) step, while the acquisition horizontal field width (‘HFW’, μm) divided by the number of pixels from the image aspect ratio defines the pixel size. For example, for the Cell #1^28^ (see Table 1) the acquisition HFW is 14.9 μm, and collected images were 6144 pixels wide, resulting in 14.9/6144 = 0.002425 μm, or 2.43 nm.

Alignment and filtering of the FIB-SEM stacks were carried out using Dragonfly (2017 version) image processing software. Upon importing the files into the software, X, Y and Z dimensions (in nm) were manually entered. If the data set representing an individual cell had been collected over several slice and view sessions, and was broken up into sections, the individual sections were combined at this stage of the analysis. The image stacks were then aligned using the *Maximization of Mutual Information* function.

Briefly, using the reference image, each subsequent image was shifted in X-Y against the reference image until the ‘mutual information’ score between the two images was at its maximum. A screen captured demonstration of FIB-SEM data alignment and filtering procedures using Dragonfly software package was deposited at figshare^27^. However, since the Z-step for all of our datasets was at 10–20 nm, which is significantly larger than the X-Y pixel size (1.95–2.75 nm) we cannot rule out the possibility of some remaining distortions following the alignment procedure.

If a cell originally had two or more sections (i.e. was a concatenated stack), the first reference slice (shown in blue in Dragonfly) was moved to align with the current slice (shown in red). Once the first slice of each section in the concatenated stack was manually aligned to the previous slice (of different stack), the abovementioned alignment steps were carried out. Once no horizontal lines were visible on either of the orthogonal views (indicating misalignment), the new data set was saved for further analysis.

Aligned stacks were then subsequently filtered using edge-preserving and smoothing filters to normalize the local contrast by simultaneously sharpening cellular membranes and gold beads as needed. A more detailed description is provided within the ‘step-by-step instruction notes’ section below.

### 3D volume segmentation

**Fig. 2:**
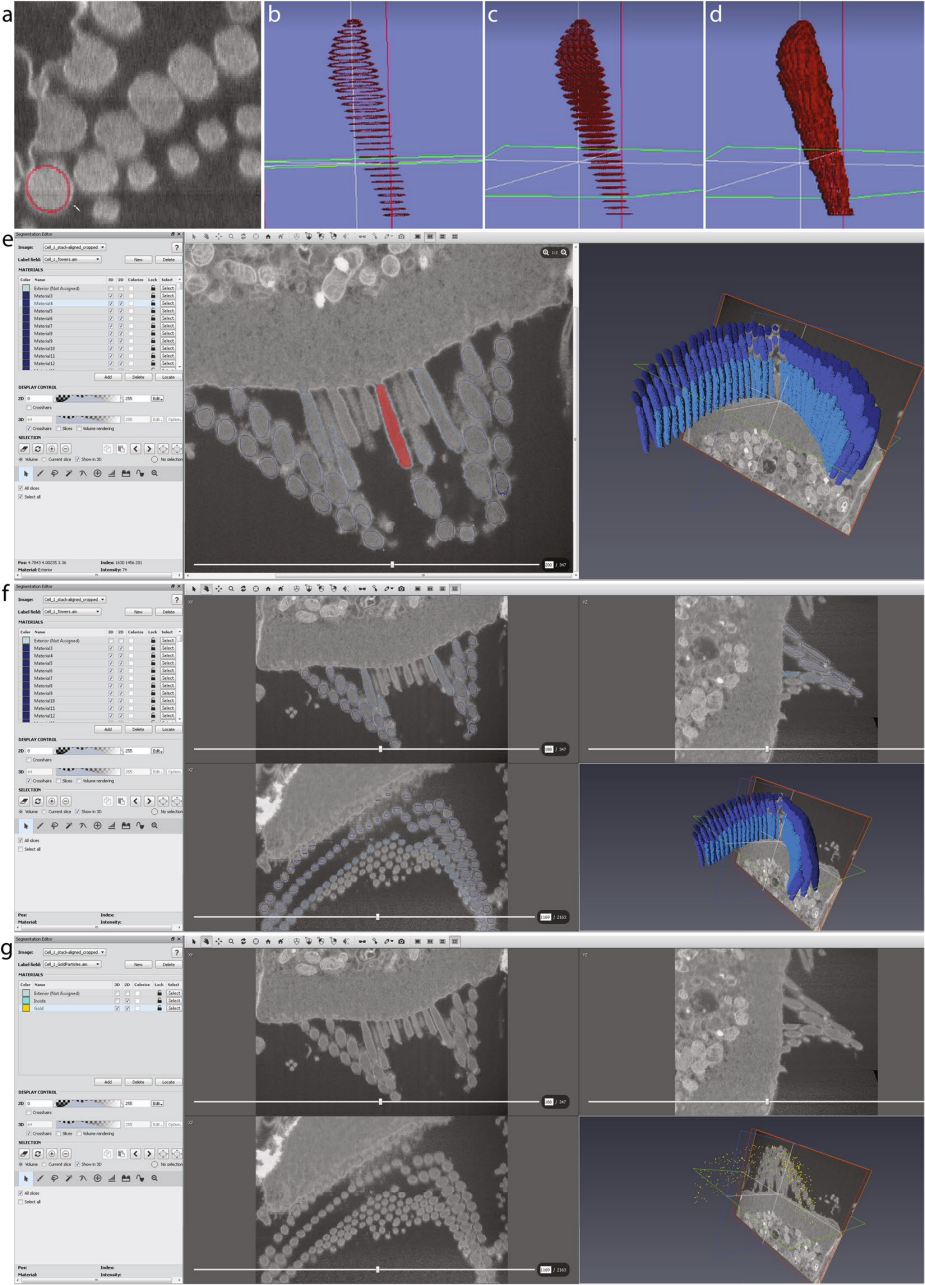
Stereocilia and gold bead segmentation workflow using Amira software package. (**a**–**d**), Stereocilia segmentation. The membrane of each stereocilium was outlined with a brush tool as shown in *a* every few sections depending on the orientation of the section through the cilium (*b*), then selections were ‘filled’ within respective section frames using the ‘fill holes’ function (*c*), and interpolated through the sections in between to segment the entire stereocilium (*d*). (**e**) Amira screen view with all voxels comprising a single stereocilium selected. (**f**) Amira screen view illustrating all stereocilia segmented within the bundle. Note only three rows of stereocilia were segmented, leaving the remaining shorter stereocilia out. (**g**) Amira screen view illustrating all gold beads segmented on the surface of the bundle.

Segmentation of the image stacks was carried out in *Amira 6.2.0*, using the XY and XZ orthogonal slice views. Upon importing the image stack, the voxel dimensions were entered once again, this time in micrometers. Once the data were loaded, a LabelField is automatically generated, corresponding to the imported stack in the ‘*Segmentation*’ tab. With a size-7 brush, the outline of each stereocilium was traced within the cell membrane on approximately 50 individual slices, each time skipping about 15–30 slices (150–300 nm) in between. Any electron dense materials outside of clearly visible stereociliary membranes (like the surface coats and occasional artifacts) were not included as part of stereocilia during segmentation. The outlines of the stereocilium made by the brush were then filled using the ‘*Fill holes - >* *All slices’* function. Next, the selection was interpolated through the skipped slices using the ‘*Selection -* > *Interpolate’* function (Fig. 2). Once a selection was made, the voxels constituting each stereocilium were assigned to a separate *‘material’*, i.e. a separate object within the volume, and the material order number reflects the ID number of each stereocilium, later used in the *MATLAB* analysis workflow. A screen captured demonstration of FIB-SEM data segmentation using Amira software package was deposited at figshare^27^.

The gold beads were segmented using a size-5 brush on the XY orthogonal view. This workflow resulted in fully segmented stereocilia, each with a unique ID number, typically corresponding to the order they were segmented during the previous step of the analysis. These ID numbers were later used to assign the row identity of each stereocilium for the *MATLAB* analysis, also used to specify whether any individual stereocilium is to be excluded from the analysis.

The Amira project can be saved in several ways containing more or less data, but only two files were essential for further *MATLAB* analysis: the *‘materials’* segmenting the volumes of each cilium (saved as *‘Towers.am’* file), and the volumes representing the gold beads (saved as *‘GoldParticles.am’*). Each file can be opened in ImageJ and viewed as a multi-page image document. Each pixel of this file has been assigned an *‘index’* value, ranging from ‘#0’ for the background material (i.e. the unused pixels), increasing up to the number of segmented cilia within the bundle.

In this study, only three rows of stereocilia were segmented, even when additional shorter stereocilia were observed. The shorter stereocilia were excluded from our analysis because their functional significance at that developmental stage is not clear, and they are not present in mature hair cells. Thus, for each dataset, a segmented bundle consisted of (1) the kinocilium, (2) the tallest row stereocilia, (3) the middle row stereocilia immediately next to the tallest row stereocilia, and (4) the short row of stereocilia, defined as shorter stereocilia immediately next to the ones from the middle row. Any stereocilia not in immediate proximity to the middle row stereocilia were excluded from the study. Sometimes the analyzed serial EM volume did not include the entire length of the stereocilium (for example, see Cell #3^44–50^), in which case the stereocilia were still segmented, but excluded from the *MATLAB* analysis as described below.

**Step-by-step Instructions: Pre- and Post-Reconstruction of 3D datasets**

I. **Create a stack**. Preferred software package – *ImageJ*.
  A. Open the raw images.
  B. Drag files (in order) to ImageJ window.
  C. Once all images are open, go to: *Image* > *Stacks* >* Images to Stack*.
  D. Save stack as TIFF format into a desired folder.
    *Note*: If the data set representing an individual cell has been collected over several slice and view sessions, and is broken up into sections, make separate stacks for each session, then combine them: *Images > Stacks > Tools > Concatenate*.
  E. Crop the stack, ideally keeping the size below 4 Gb.
    *Note:* for deposition purposes, all data sets deposited with this submission were built into a stack to enable their quick access and view in the browser using the “detailed view” link to the repository’s online image viewer window.
II. **Determine the voxel size and the Z step**.
  A. Voxel Size.
    1. Open one of the images in the Notepad application, or in MS Word.
    2. Scroll to the bottom of the file, search for “PixelSize” → X unit = “m” = “2.43E-09”. The value reflects the pixel size in X and Y.
      *Note:* Alternatively, open the *‘Projectlog.log’* file, and find the acquisition horizontal field width (“HFW”, μm), and divide by the number of pixels in the image aspect ratio to calculate the pixel size. For example, for Cell #1, the acquisition HFW is 14.9 μm, and collected images were 6144 pixels wide, resulting in 14.9/6144 = 0.002425 μm, or 2.43 nm (Table 1). All *‘Projectlog.log’* files were deposited with corresponding datasets, and can be found in the accompanying compressed (zipped) folders.
  B. Z step.
    1. For Z, open the *‘Projectlog.log’* file and search for “targetthickness”. The value reflects the Z milling step size in meters: “1.5E-08” is 15 nm.
III. **Aligning and Filtering**. Preferred software package – *DragonFly*.
  A. Open Dragonfly application, and go File > Import Image Files > Add… > (choose stack) > Next.
  B. For Image Spacing (in nm), convert x, y, and z dimensions to nm, then click *Finish*
    1. Example: X, Y = 2.43… E-09 m = 2.43… nm.
    2. Example: Z = 1.50 E-08 m = 15.0 nm.
  C. *Note*: If the data set representing an individual cell has been collected over several slice and view sessions, and is broken up into sections, follow the steps for each individual section.
  D. Alignment.
    1. On “Data Properties and Settings” panel, right-click on stack *file* > *Data Slices Registration*.
    2. On Slice Registration Window:
      - If cell originally had only one section, continue by: Registration method > Mutual info > Settings > Maximum translation: small > Apply.
        *Note.* Mutual Info:
        Is a measure of mutual dependence between the two variables.
        Given a reference image, and a second image which needs to be put into the same coordinate system as the reference image, the image is rotated and translated (in X-Y) until the mutual information between it and the reference image is maximized.
        Considers all voxels in the images to be registered to estimate the statistical dependence between corresponding voxel intensities.
        *Note.* Maximum translation:
        Determines the maximum translation that will be allowed between two slices.
        Small setting limits to 0.5% of the size of the image.
      - If the cell originally had two or more sections (concatenated stack), first: Registration method > Manual → move reference slice (blue) to match the current slice (red).
        1. Once the first slice of each section in the concatenated stack is manually aligned to the previous slice (of the different stack), follow Mutual info alignment steps as above (a).
      - Go through the aligned stack, and make sure there are no horizontal lines on either orthogonal view (indicating misalignment).
        1. Once satisfied with the result, click *Create New Data Set > OK*.
  E. Filtering. The Histogram balancing, Unsharp and Mean shift filters were found most useful in processing the datasets. Also could be considered the Slope map; CLAHE; Sobel; Median; Bilateral; Laplacian; and Gaussian filters.
    1. To apply a filter, run Tools > Image Processing Toolbox > Import Image Files > (select stack/concatenated stack) > (input voxel dimensions in nm, as in step III).
    2. If brightness/contrast varies between slices within the stack, consider using Histogram Balancing filter, and choose reference slice. Note: Histogram balancing filter spreads out the most frequent intensity values in an image. The equalized image has a roughly linear cumulative distribution function.
      - Reference slice - a representative slice with ideal brightness/contrast ratio that will be applied to ALL slices.
      - Settings found useful: unit: 8 (default) > reference slice: __ (enter slice number).
    3. If the stack appears blurry (slight ambiguity in details), consider using Unsharp filter *Note: Unsharp filter* sharpens an image by subtracting a blurred version of itself from the original.
      - May make more fuzziness to stack, follow up with step 4 (below).
      - Settings found useful: unit: 8 > options → kernel: 3D; 3 > standard deviation: 1.00; unsharp factor: 1.0.
    4. If background noise is an issue, consider using additional filters, such as:
      - Median filter. Settings found useful: unit: 8 > kernel: 3D; cube; 3.
      - Mean shift filter. Settings found useful: unit: 8 > kernel: 3D; cube; 3.
      - Bilateral filter. Settings found useful: unit: 8 > kernel: 3; *σ* color = 1.00; *σ* spatial = 1.00.
      - Gaussian filter. Settings found useful: unit: 8 > kernel: 3D; 3; standard deviation: 1.00.
      - Sobel filter.
      - Contrast Limited Adaptive Histogram Equalization (CLAHE) filter. Settings found useful: unit: 8 > clip: 0.01 > kernel size: 117 > Bins: 258.
    5. Once desired filters have been applied, go *Output > Apply*. Wait for filters to load and apply, then close image processing window.
      - Save stack by going to Data Properties and Settings Panel > (right-click) > Export Images… > (select location) > (check box “Output all images into one file so it can form an image stack) > Save.
IV. **Segmentation**. Preferred software package – *Amira (FEI)*, version 6.2.0; *Avizo (FEI)*.
  A. Start-up and Data Import.
    1. Open Amira application, and go to Open Data > (select filtered stack) > Out-of-Core Data window will appear.
      - If 200 GB of hard drive space is available, select “*Read complete volume into memory*”; if not, select “*Convert to Large Data Access (LDA) format*”.
    2. Image Read Parameters window will appear, input voxel size dimensions in microns.
      - Example: X, Y = 2.5849…E-09 m = 0.0025849… μm.
      - Example: Z = 1.50 E-08 m = 0.015 µm.
  B. Segmentation Editor.
    1. Once data has been loaded, select the “*Segmentation*” tab. This is the Segmentation Editor where the stack will be reconstructed.
      - A LabelField will automatically be generated that corresponds to the imported stack.
    2. To add a material (which will be for one cilium), select “*Add*” and a material field will appear.
  C. Segmentation procedure.
    1. From there, on the Selection window, press the paintbrush tool. This will be used for segmenting the cilia.
    2. Change the size of the brush to 7 if working in 1:1 zoom.
    3. To segment, trace the circumference/perimeter of the cilium and repeat every 2–3 slices (for XY view) or every 15–30 slices (for XZ view).
    4. Add selection as a ‘new material’.
    5. Go to *Segmentation > Fill holes > All slices* to fill the area within the selection on all slices with outlined membranes.
    6. Go to *Selection > Interpolate* to select the voxels in between the selected planes, and add selected voxels to the same ‘material’.
    7. OHC segmentation.
      - For tall and medium stereocilia row, use XY orthogonal view.
      - For small stereocilia row, use XZ orthogonal view.
        *Note:* All stereocilia are within the same LabelField, but each cilium represents an individual material.
    8. Gold beads. *Note:* only gold beads near the segmented cilia were segmented.
      - Use XY orthogonal view with brush size 5 in 1:1 zoom.
      - Only identify the brightest/most obvious gold beads; skip repeats in following slices.
        Note: use different material than segmented cilia; each gold bead added to the SAME material.
V. **Post-segmentation** - Saving data and tables.
  A. To save the Amira project (LabelFields + stack), go File > Save Project As… > Select “Minimize Project Size” > (select folder to save) > Save data type as “Amira Project and data files [pack & go] (.hx)”.
  B. If you wish to save only the LabelFields (segmented rows + gold), the only files required for the MATLAB code analysis, then on the Project tab select the LabelField desired > right-click > Save Data As > Save data type as “Amira RLE (.am)”.
  C. After segmentation of stereocilia bundles and cuticular plate (including the gold beads) is complete, turn on the 3D volume rendering view on the Segmentation tab.
  D. Open an Excel file to specify which stereocilia are connected to each other.
    1. Values to be placed in the excel spreadsheet are the “intensities” of the cilia, and reflect their ID numbers.
      - To find stereocilia ID numbers:
        1. Open LabelField in Amira, and change Image Source (on Segmentation Editor) to “No Source”.
        2. Hover over each cilium with the cursor, and look at the bottom-right corner “Selection” window to get the intensities.
          Note: All LabelField ‘.am’ files can also be opened in ImageJ, in which case the ID number of each cilium is the “index” value displayed on the bottom line of the main window of ImageJ upon positioning the cursor over the cilium of interest. For all background voxels, the “index” value is set to zero (assigned to any material).
    2. Order of rows within the spreadsheet is expected to be as follows: Kinocilium (identified as row 1 towers by the *MATLAB* scripts), tallest row stereocilia (row 2 towers), middle row stereocilia (row 3 towers), and shorter row stereocilia (row 4 towers). Instructions on how to prepare the two necessary tables for the *MATLAB* analysis (*LinkTable.csv* and *TowerUsage*.csv) are provided within the Volume analysis section below, accompanied by Tables 4 and 5.

**Table 4:** Summary of tower connections determining the most likely MET direction, as in *‘LinkTable.csv’* file.

Towers	ID numbers:				
Row 1 (kinocilia)	0	0	109	0	0
Row 2 (tallest row stereocilia)	20	19	18	17	16
Row 3 (middle row stereocilia)	56	55	54	53	52
Row 4 (short row stereocilia)	92	91	90	89	88

**Table 5:** Binary table instructing which towers shall be included in the analysis, as in *‘TowerUsage.csv’*. The kinocilium and all stereocilia listed in Table 4 are included in the analysis.

Towers	Inclusion				
Row 1 (kinocilia)	0	0	1	0	0
Row 2 (tallest row stereocilia)	1	1	1	1	1
Row 2 (middle row stereocilia)	1	1	1	1	1
Row 2 (short row stereocilia)	1	1	1	1	1
VI. Folders and files used for *MATLAB* analysis.
  A. Save the cilia and gold LabelFields only, using Amira, as separate.am files
  B. the cilia LabelField as ‘Towers.am’ and the gold bead LabelField as ‘GoldParticles.am’
  C. Name the stereocilia connection table “LinkTable.csv” and the tower usage stereocilia table (a binary table excluding incomplete cilia as 0 and including the complete cilia as 1) as “TowerUsage.csv”.
    1. “LinkTable.csv” allows the MATLAB algorithm to determine the orientation of each cilium based on provided information on which cilium is likely connected with which neighboring one with a “tip link” to determine each stereocilia’s mechanosensitive direction. The text below refers to the ‘north’ direction for each stereocilium, which is a vector pointing 180° away from the mechanosensitive direction.
    2. “TowerUsage.csv” table allows one to exclude individual stereocilia from the analysis and was used to exclude some stereocilia that were only partially included in the dataset. For example, see Cell#350 and the test Dataset 774, described in a greater detail within the Technical Validation section below.
    3. Save both tables as ‘csv’ files.
  D. It is important to get the correct file names and formats for the script to successfully execute the *MATLAB* analysis step.

### 3D-structure reconstruction, surface rendering

**Fig. 3:**
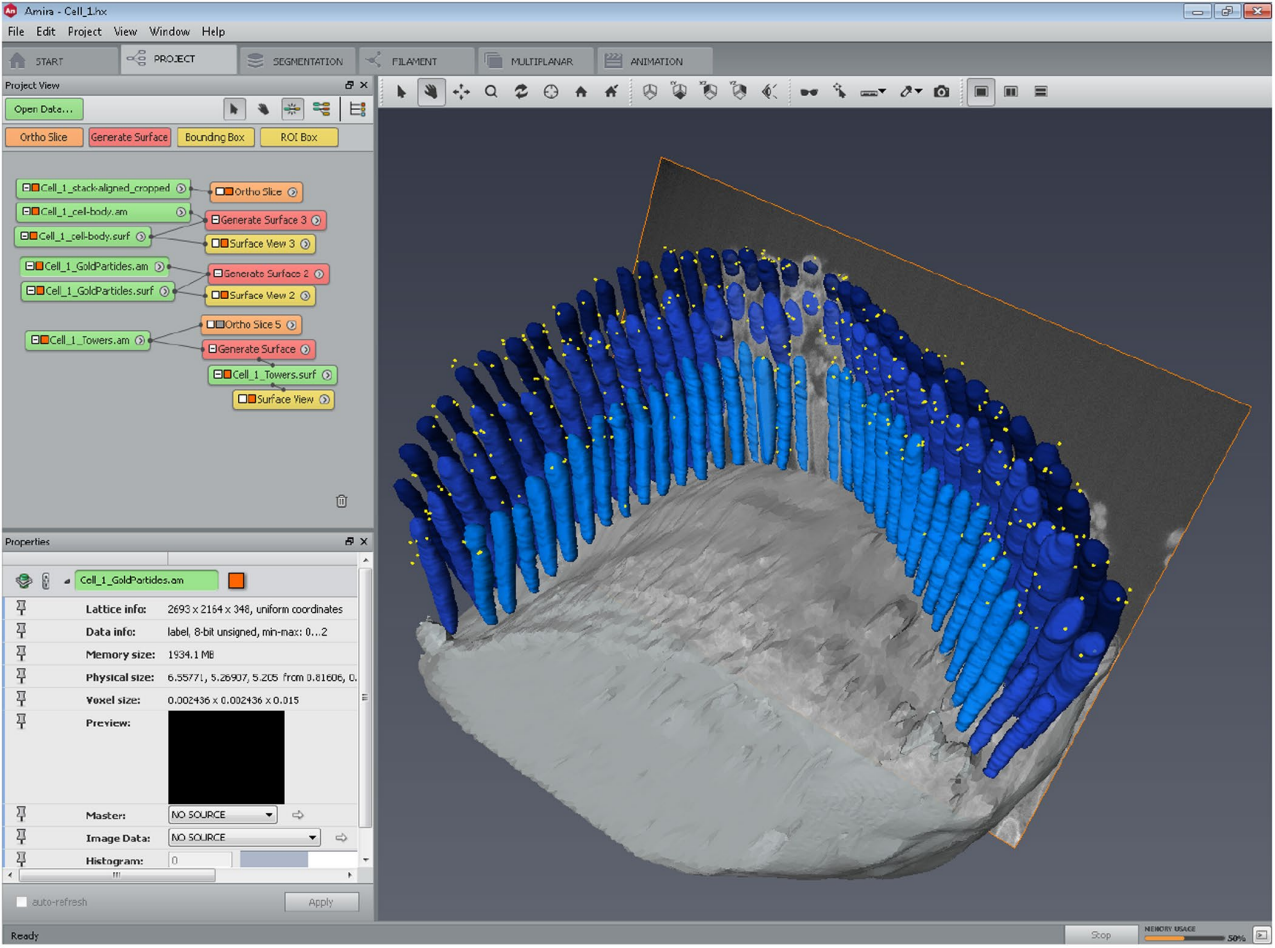
Screen capture of Amira project at the step of surface rendering. Following segmentation, each stereocilia bundle used in this study was subsequently rendered using the *‘Generate surface’* function of Amira for visualization purposes. The results are also provided in a movie illustrating 3D localization of PKHD1L1 in immunogold labeled outer hair cell stereocilia bundles using FIB‐ SEM, available at figshare^27^.

An additional step was taken to generate the surfaces of the segmented volumes for demonstration purposes. This step has been carried out in Amira, by generating the surfaces of all objects selected for the illustration, and rendering a movie. The color coding used in the rendered movies was only intended for illustration purposes, presenting stereocilia of each row in a distinct shade of blue (Fig. 3). The row identities were kept consistent with the ‘*LinkTable.csv’* file used in *MATLAB* analysis.

In order to generate and edit the surface on the ‘*Project tab’* the created LabelField was selected, then *‘Generate Surface’* module was connected to the LabelField. Executing the ‘*Generate Surface’* function takes some time, depending on the size of the model. When creating the ‘*Generate Surface’*, the degree of smoothing applied to each surface was modified as needed. This is particularly important, as in some cases extensive smoothing could cause some small or thin structures to disappear. A smoothing type port ‘*uncontrasting smoothing’*, set to smooth level 5 was used to visualize the cell body. It created a smoothed surface with few constraints and some loss in details. For the LabelField ‘*Towers.am*’ containing the cilia segmentation, and the LabelField ‘*GoldParticles.am*’ containing the gold bead segmentation, no smoothing was applied. For these files, each surface appeared as a block rendering, preserving the highest level of detail as originally segmented. The*’Surface View’* module was then used to visualize the entire model in the Amira viewer window. For ‘*Towers.am*’ Draw Style’*transparent’* was applied to superimpose the surfaces using a’*base transparency level’* set to 0.3. The best representation of stereocilia was achieved by selecting such additional options as ‘*Fancy alpha’*, ‘*Sorting’*, ‘*Both faces’*, ‘*Vertex normal’* in the Draw Style port of the’*Surface View’* window. For ‘*GoldParticles.am*’ the Draw Style’*outlined’* was applied to superimpose surfaces with ‘*line width 1’* and outline color set to’*yellow’*. The best representation of gold beads was achieved by selecting additional options such as ‘*Specula’, ‘Opaque’, ‘Both faces’, ‘Triangle normals’* in the Draw Style port of the’*Surface View’* window. For ‘*Cellbody.am’* the Draw Style’*transparent’* was applied to superimpose the surfaces, with’*base transparency level’* set to 0.4, and with the default options ‘*Texture’*, ‘*Fancy alpha’*, ‘*Sorting’*, ‘*Both faces’*, ‘*Triangle normal’* in the Draw Style port of the*’Surface View’* window.

The output result is summarized in a movie illustrating 3D localization of PKHD1L1 in immunogold labeled outer hair cell stereocilia bundles using FIB‐ SEM deposited at figshare^27^, and published with permission from the original study^17^. These were saved as*.hx* ‘*Amira Project and data files [pack & go]*’ and are available for download as compressed (zip) folders as part of the submitted datasets^34,42,49,58,65,70^.

### Volume analysis

The post-segmentation analysis was performed using a custom *MATLAB* algorithm^75^, aiming to quantify the distribution of gold beads in 3D. The scripts are deposited with this submission, are available for download (10.7295/w9cil50728), and a detailed, step-by-step description of the algorithm is outlined below.

Stereocilia registration and gold bead quantification were performed in *MATLAB*. Due to the tower-looking nature of stereocilia and kinocilia, we refer to them as towers for the volume analysis. Three scripts were generated to carry out the analysis: (1) *‘towersOfGold’; (2) ‘togDisplayResults’; and (3) ‘togAggregateResults’*.

These scripts expect a data organization with the folders as shown below:

All_Cells

    Cell3^50^

         GoldParticles.am

         LinkTable.csv

         Towers.am

         TowerUsage.csv

    Cell4^59^

         GoldParticles.am

         LinkTable.csv

         Towers.am

         TowerUsage.csv

         In such a case, the analysis steps are:

1. Analyze individual cells by running *‘towersOfGold’* independently on subfolder ‘All_Cells/Cell3’ and ‘All_Cells/Cell4’;
2. To visualize the output of the analysis on an individual cell, run *‘togDisplayResults’* on the corresponding subfolder, for example on ‘All_Cells/Cell3’;
3. To aggregate the outputs of the analysis for all cells in All_Cells, run *‘togAggregateResults’* on the parent folder All_Cells.

As illustrated, input data for each cell consist of 4 files: *‘GoldParticles.am’, ‘Towers.am’, ‘LinkTable.csv’*, and *‘TowerUsage.csv’*. The *‘.am’* files are segmentation volumes obtained with Amira, and correspond to tower labels as well as gold bead masks. *‘LinkTable.csv’* contains annotations of the neighborhood relationships between towers. Because the hair cell stereocilia bundle is a polarized structure, and the MET axis varies between the pairs of stereocilia within the bundle, we reasoned that the individual position of each stereocilium with regards to the MET axis should be taken into account when determining the average distribution of the gold beads across the population of stereocilia. Although the position of the tip link mediating the MET is the best predictor of the MET axis for any given pair of stereocilia, our FIB-SEM datasets did not visualize the tip links well enough to rely on their position when determining the MET axis. Instead, we developed an alternative approach in which each stereocilium within the bundle was assigned its ‘most likely’ connection with stereocilia from the neighboring rows based on their position.

**Fig. 4:**
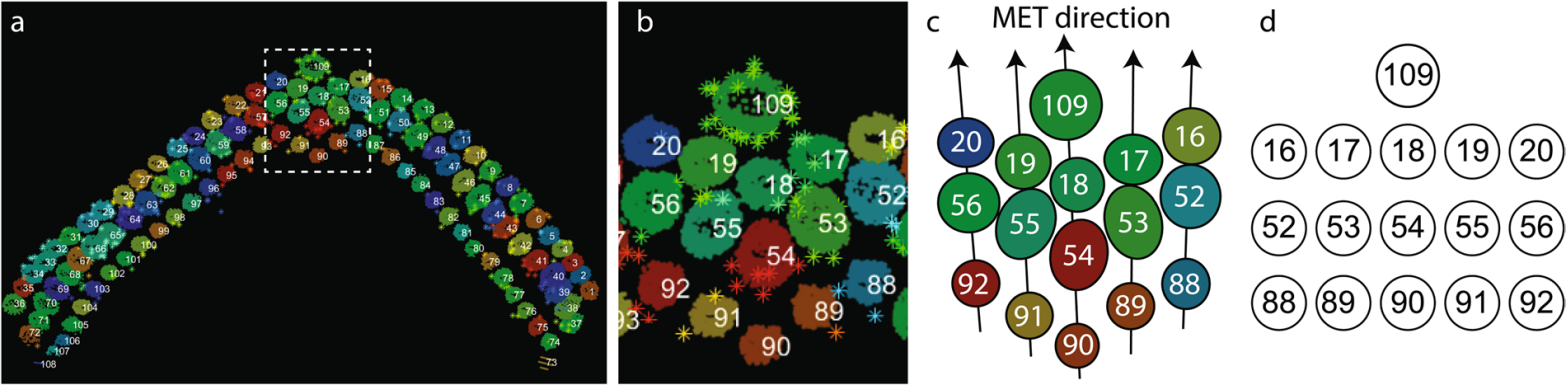
Schematic diagram illustrating the process of assigning connections to determine the MET direction for each column of stereocilia within the bundle. (**a**) Top view of OHC stereocilia bundle segmentation volume. Each stereocilium and the kinocilium is labeled with its ID number. White dashed box outlines the area shown in *b*. (**b**) A higher magnification view of the central subset of stereocilia. (**c**) A schematic representation of stereocilia arrangement from *b*. Black arrows passing through stereocilia and the kinocilium represent the most likely MET direction for each set (*‘column’*) of stereocilia. (**d**) A schematic representation of the same set of stereocilia and the kinocilium as it would appear in the “*LinkTable.csv*” file, also presented in Table 4.

The process of assigning stereocilia within the ‘*LinkTable.csv*’ file is illustrated in Fig. 4 using a subset of stereocilia from Cell#3^50^, while the final result is presented in Table 4. In this example, tower 109 in row 1 (the kinocilium) is immediately behind tower 18 in row 2, which in turn is flanked by towers 17 and 19 (all tall row stereocilia). Zeroes in such table are used as placeholders, and do not correspond to any tower. The structure and content of Table 4 is similar to the corresponding ‘*LinkTable.csv*’ file, although the latter omits the column and row headings.

*‘TowerUsage.csv’* is a binary table, of identical size as ‘*LinkTable.csv*’, indicating which towers shall be considered during analysis. For example, a binary table corresponding to towers presented in Table 4 is summarized in Table 5. The structure and content of Table 5 is similar to the corresponding *‘TowerUsage.csv’* file, although the latter omits the column and row headings.

In the excerpts above, all towers are used, but there are cases where some are excluded due to inaccuracies in segmentation or data collection (for example see Cell#3^50^, used for the analysis below).

**Fig. 5:**
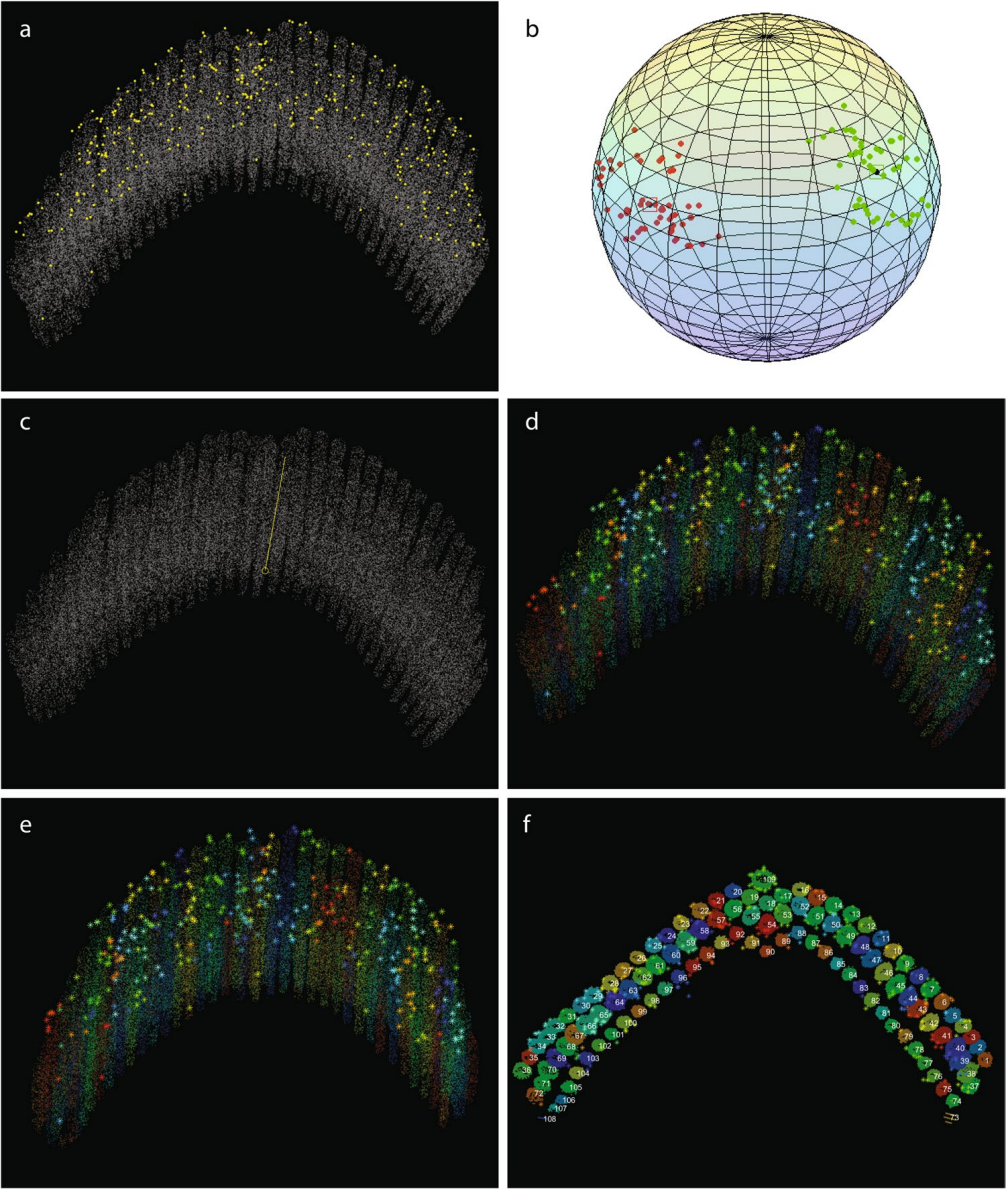
Output figures generated by the *‘towersOfGold’* script for Dataset 3 (Cell #3^44–50^) reflecting the analysis workflow. (**a**) After the dataset was read and resized (optional), borders of tower masks were sampled, centroids of gold beads computed and displayed. (**b**) Each tower’s principal directions are computed and displayed: red and green dots represent tower directions (after a random flip); black dots (surrounded by squares) represent direction cluster centers, obtained via mean shift. (**c**) ‘Upward’ direction (identifying the tips of stereocilia) are determined and displayed. (**d**) Gold beads are associated with the towers using either ‘nearest neighbors’ or ‘distance transform’ function. (**e**) Towers and all associated gold beads are aligned to the upward direction, positioning all towers parallel to each other. (**f**) Top view of towers, along with tower IDs, after data transformation setting up the upward direction as [0 0 1], and the bottom plane set to z = 0. Same image as in Fig. 4a.

**Fig. 6:**
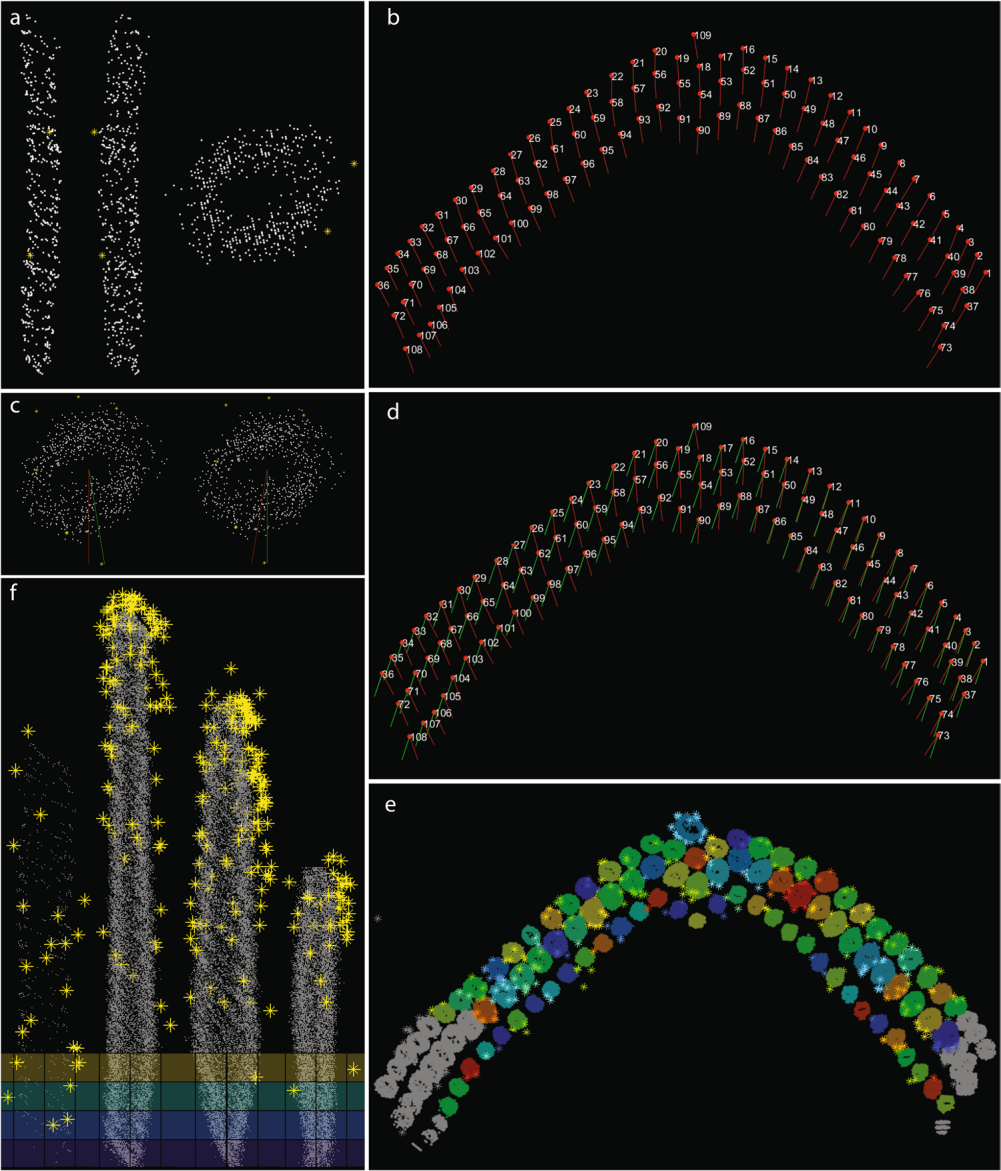
Output figures generated by the *‘towersOfGold’* script for Dataset 3 (Cell #3^44–50^), continued. (**a**) Perpendicular views (right, front, top) of a single tower, and the gold beads associated with it. (**b**) Azimuth direction for each individual tower computed using the ‘*LinkTable.csv*’ table, and displayed as a red vector pointing north from the center of each tower. The ‘north’ direction for each tower is defined as a vector pointing 180° away from its mechanosensitive direction. (**c**) Azimuth transform for a single tower (top and bottom views); the red line points north before the transform, while the green line points to a common north (for all towers) after the transform. (**d**) Azimuth transforms computed for the entire bundle. *Red*, the original azimuth direction; *green*, the resulting azimuth direction after the transform. (**e**) Azimuth transform applied to all towers and associated with gold beads. Using the *‘TowerUsage.csv’* table, all towers excluded from further analysis are displayed in gray. (**f**) All towers included in the analysis and their associated gold beads were registered to a common, template tower for each row, and the convex hull of aggregated towers computed. Based on the identity specified for each tower, towers were divided into four groups: row1 (kinocilium), row 2 (tall row stereocilia), row 3 (middle row stereocilia), and row 4 (short row stereocilia) towers.

*‘towersOfGold’* script outputs two types of data: figures and tables. After running the *‘towersOfGold’* script on All_Cells/Cell3, for example, two folders will be created in folder corresponding to Cell3, subsequently named *‘Figures’*, and *‘Tables’*. In *‘Figures’* folder, live *MATLAB* plots (*fig* format) are saved, also shown on Figs. 5 and 6. The figures can be opened in *MATLAB* upon completion of the analysis. In *‘Tables’* folder, a number of tables are saved, containing:

- row-aggregated gold bead coordinates (X, Y and Z), entitled *AgrGPRow1.csv, AgrGPRow2.csv* etc.;
- row-aggregated tower convex hull coordinates (*AgrTwrRow1.csv, AgrTwrRow2.csv*, etc.);
- heights and radii of every tower within the same row (*HtRadRow1.csv, HtRadRow2.csv*, etc.);
- average heights and radii of towers for each row (*AvgHeightAndRadius.xls*).

Tables can be visualized by running *‘togDisplayResults’*.

Finally, *‘togAggregateResults’* script collects and aggregates results of *‘towersOfGold’*, as well as further computing ‘cylinder’ and ‘polar’ histograms of gold beads. In this dataset example, *‘togAggregareResults’* applied to All_Cells would output such analysis in a number of images and tables under All_Cells. Details on this script are provided below following the detailed workings of *‘towersOfGold’* script.

*Script 1. ‘towersOfGold’*.

The script analyzes one cell at a time. The following parameters need to be set:

dataPath: path to the folder containing the.am files as well as ‘*LinkTable.csv’* and ‘*TowerUsage.csv’;*

resizeFactor, default 1/4: the*.am* volumes will be resized by this amount to speed-up computations;

borderSamplingFactor, default 1/10: not all tower border voxels are necessary for some computations and visualizations, thus we only use a random sample subset;

gold2towerAssociationMethod, default ‘NN’, optionally ‘DT’: to associate gold beads to towers, there are two possibilities, *‘nearest neighbors’*–which is faster–or *‘distance transform’*. Nearest neighbors algorithm was used for our data analysis.

nNearestNeighbors, default 10: when gold2towerAssociationMethod = *‘NN’*, this is the ‘number of nearest neighbor’ parameter;

minDistThr, default 100 (in micrometers). A threshold distance from the gold bead to the nearest tower. If positioned further than this threshold, the bead is excluded from analysis.

The *‘towersOfGold’* script performs the following steps:

1. Setup output folders.
2. Read and resize data, sample border of tower masks, and compute centroids of gold bead blobs display Fig. 5a.
3. Compute tower principal directions via PCA (principal component analysis); due to ambiguity on the sign of the output directions (consequence of ambiguity in the sign of eigenvectors), we randomly flip their signs, thus forcing about half directions to point to each end of the lines crossing the towers longitudinally. This way the set of principal directions form two diametrically opposed clusters in a 3D sphere, with nearly the same number of elements each, which increases robustness of the mean-shift clustering algorithm in the next step. Display Fig. 5b upon completion of the step.
4. Determine ‘upward’ direction from the outputs of Fig. 4b; this is defined as the direction such that the minimum projection of the towers data has the smallest standard deviation; i.e., it is the direction opposite the one where the tower extremities are approximately aligned to a plane. Direction candidates are obtained by clustering of directions from step 3, which is performed using mean-shift in a sphere^76^. Mean-shift outputs two nearly opposite directions. Display Fig. 5c upon completion of the step.
5. Associate gold beads to towers using either ‘nearest neighbors’ or ‘distance transform’, and display Fig. 5d.
6. Align towers and gold beads to upward direction, by rotating them around their center of mass; rotation of a point about a vector in 3D by a certain angle is done using the Rodrigues rotation formula^77^, then display Fig. 5e.
7. Transform data such that upward direction is [0 0 1], and the bottom plane is z = 0. Display Fig. 5f showing the top view of towers, along with tower IDs, after transformation. Figure 6a shows perpendicular views (right, front, top) of a single tower, as well as its associated gold beads.
8. Compute azimuth direction for individual towers, according to data from ‘*LinkTable.csv*’ then display Fig. 6b.
9. Compute azimuth transforms for individual towers / gold beads, so that ‘ahead’ direction is [0 1 0]. Display Fig. 6d, showing the original azimuth direction (in red) and the final azimuth after transform in green. Figure 6c shows the azimuth transform for a single tower (top view); the green arrow points north before the transform, while the red arrow points to north after the transform.
10. Apply azimuth transform to all towers and associated with them gold beads, taking *‘TowerUsage.csv’* into account. Display Fig. 6e.
11. Compute average height/radius per row of towers.
12. Register tower rows to common (template) tower using the average height/radius for each row of towers then display Fig. 6f.
13. Compute convex hull of aggregated towers; save convex hull data and gold beads locations to disk.

*Script 2. ‘togDisplayResults’*

This script visualizes the output of the analysis for an individual cell. To execute the script, run *‘togDisplayResults’* on the corresponding folder, for example, All_Cells/Cell3, and figures as shown on Figs. 5 and 6 will be generated and displayed.

*Script 3. ‘togAggregateResults’*

**Fig. 7:**
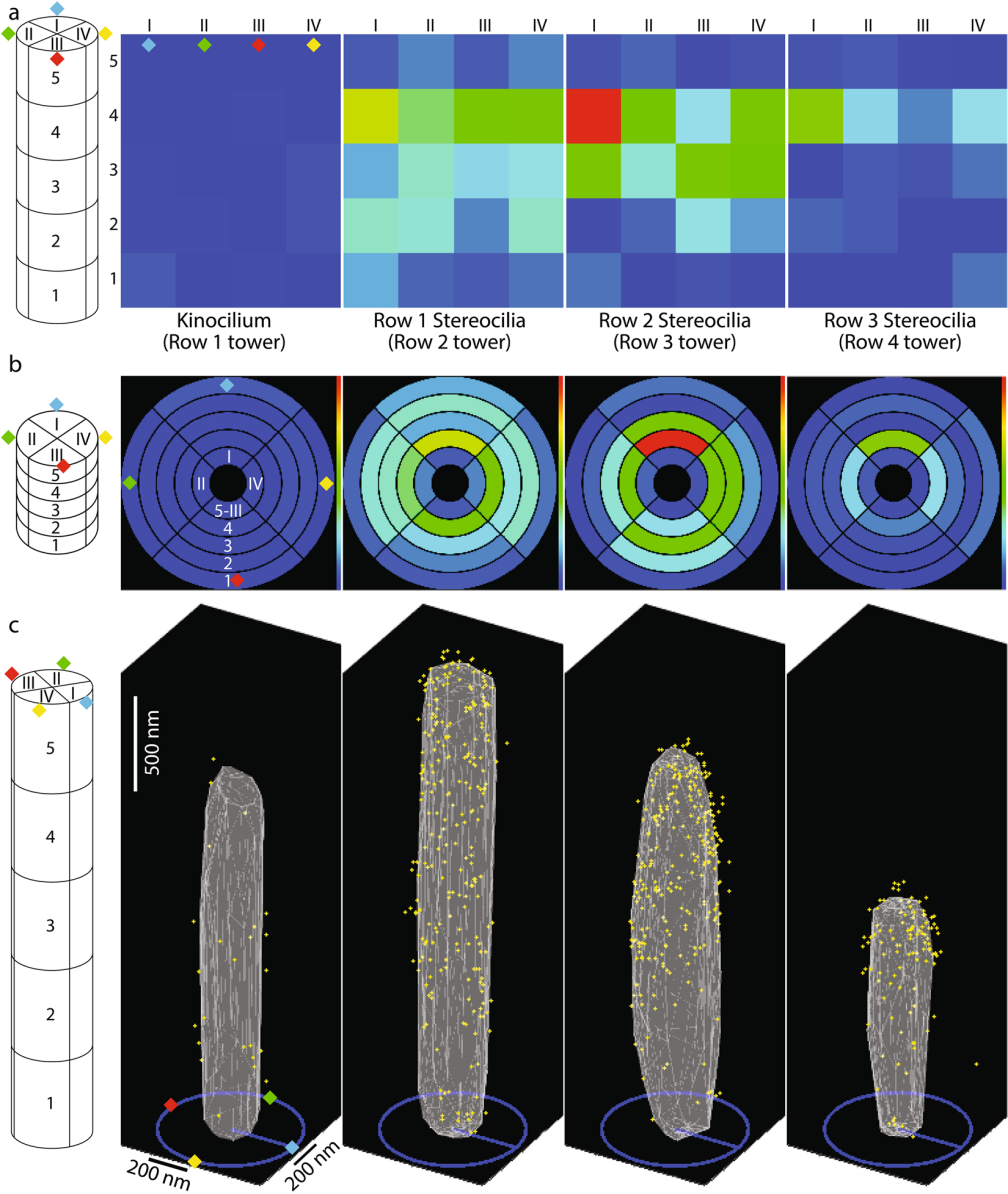
Examples of output figures generated by *‘togAggregateResults’* script. **(a,b)** The output figures display the gold bead distribution within a defined number of segments, which were further subdivided into a defined number of radial sectors. For this illustration, five segments along the length of stereocilia were chosen, numbered from 1 to 5 from the proximal end of stereocilia to its distal end, respectively. The most distal segment (#5 in this figure) always represents the volume *above* the tip of stereocilium. Each segment was further subdivided to four radial sectors (azimuth bins), numbered I, II, III and IV (blue, green, yellow and red diamonds, respectively), as shown on schematic diagrams on the left of all panels. Both panels, *a* and *b* represent the same data, and are displayed as a heat map. In *a*, the surface of the cylinder is represented in a ‘side view’ as a rectangle of the lateral surface of the tower. In *b* however, the surface of a cylinder is represented as a ‘top view’ arrangement of concentrically positioned rings, where the outermost ring is of the most proximal segment, while the innermost ring is of the area above the tip of stereocilium. (**c)** A 3D cumulative distribution map of gold beads. Although a specific view is shown on this panel, the actual figure generated by MATLAB is a 3D plot, and can be manually positioned at any angle. The schematic diagram on the left summarizes the actual orientation of the cilia in reference to the panels *a* and *b* using the blue, green, yellow and red diamonds. Yellow dots represent gold beads, while gray triangles represent the ‘average’ ciliary surface triangulated from the cumulative collection of surface coordinates pooled from all stereocilia analyzed.

This script aggregates results of ‘*towersOfGold’* inside a folder. In the data exemplified above, after running *‘towersOfGold’* on All_Cells/Cell3 and All_Cells/Cell4, *‘togAggregateResults’* should be deployed on All_Cells. The parameters to be set are:

dataPath: path to ‘superfolder’ containing analysis tables;

viewDirection, default [90 0]: azimuth and elevation parameters of the viewing angle of the final 3D plots; for example, a top view would be [90 90], whereas a side view would be [90 0];

backgroundColor, default ‘black’: background color of plots;

gpMarker, default ‘*‘: marker corresponding to gold beads on 3D plots;

gpMarkerSize, default 1: gold beads marker size;

nAzBins, default 4: defines the number of azimuth bins in gold beads histogram (radial sectors). The sectors are indexed counterclockwise, as viewed from the top, starting with the section pointing ‘north’, i.e. [0,1,0], labeled by a blue diamond on Fig. 7a,b, representing an example of a tower divided into four sectors. The mechanosensitive direction is defined by a vector running from the blue to the red diamond.

nHtBins, default 5: defines the number of bins along the tower in gold beads histogram; the last bin corresponds to gold beads positioned above the tower (in Z);

towersAsPointClouds, default false: when false, 3D plots show towers as triangulated meshes of their convex hulls (Fig. 7c), as opposed to the convex hull points themselves when true (Fig. 6f);

trMarkerSize, default 1: when towersAsPointClouds = true, this sets the marker size of the tower points in 3D plots;

The main outputs of the script are polar/cylinder histograms of gold bead counts per height bins (referred to as segments) and azimuth sections (referred to as radial sectors), saved as*.xls* tables. Some visualizations are also displayed and saved as*.fig* and*.png* files. Figure 7 illustrates some of the outputs of the script.

In this study, the kinocilia were excluded from the volume analysis due to a low number of observations (n = 6, one per stereocilium bundle), while all three rows of stereocilia were analyzed, and data presented for each row of stereocilia as a cumulative 3D plot. This work includes results from 197 tallest row stereocilia, 197 middle row stereocilia, 193 shorter row stereocilia, and 2185 gold beads associated with them.

To quantify the gold bead distribution at the surface of stereocilia as shown on Fig. 1m,n, further analysis was performed using the results of the *MATLAB* script. Instead of dividing stereocilia into an equal number of bins, we instead chose to assign to each row of stereocilia a number of bins that would divide the stereocilium into segments ~200 nm tall, always subdividing them to four radial sectors (anterior, posterior, and two lateral). Based on the average height of stereocilia calculated for each row, the number of segments for the tallest row stereocilia resulted in 12, plus an additional segment to include the gold beads above the tip of stereocilium. Similarly, the middle row stereocilium was divided to 10 + 1 segments, and the short row stereocilia were divided into 6 + 1 segments. The gold bead count of the most distal segment (#13, #11 and #7 for the tallest, middle and shortest stereocilium, respectively) representing the volume *above* the tip of stereocilium was added to the gold bead count of the corresponding segment below, assigning these gold beads to the very tip segment of stereocilium. The surface area of each segment was calculated using the known average diameter of stereocilia and the height of the segment for each row, which was in turn calculated as the average height of stereocilia of that row divided by the number of segments used. Normalized labeling density was then calculated as the gold count per segment area per cilium, for each stereociliary sector, and presented as shown on Fig. 1n. In addition, a movie illustrating the 3D cumulative distribution map of anti‐PKHD1L1 immuno‐gold beads from six mouse OHC stereocilia bundles (postnatal day 4) was deposited at figshare^27^. The movie, and the Fig. 1n represent the same data, and both are published with permission from the original study^17^.

## Data Records

Each dataset represents a set of raw image files and all related intermediate data sets derived from it, and are associated with the same outer hair cell. Some of the main parameters for each cell and related dataset are summarized in Table 1. Although the FEI Slice and View software package generated individual TIFF files for each section of the serial EM data set, the images were combined into a single multipage TIFF file prior to deposition at *The Cell Image Library* repository. This was done to promote easy data access, and allow for their online preview within the browser window using the “detailed view” link embedded within each entry.

### Dataset 1

Includes the following files, all related to Cell #1 of the current study. The numbering order of the files reflects the order of operations performed during the analysis. Group link: http://cellimagelibrary.org/groups/50680

*1.1_Cell_1_and_Cell6_Raw_Image_Stack*^28^ is a multipage TIFF file consisting of 388 individual EM sections acquired at 15 nm milling step, resulting in 2.43 × 2.43 × 15 nm^3^ voxel size. This file also contains the raw data related to Cell#6.

*Note:* The data acquisition session was interrupted after frame #161 to adjust the imaging area, resulting in a large shift on subsequent frame #162.

*1.2_Cell_1_Stack_Aligned*^29^ is a multipage TIFF file containing aligned images of Cell#1. The file contains 387 images.

*1.3_Cell_1_Stack_Cropped*^30^ is a multipage TIFF file containing only the relevant volume of Cell#1, cropped to reduce the file size. The file contains 387 images, as the plane #1 of the original stack was removed.

*1.4_Cell_1_Stack_Filtered*^31^ is a multipage TIFF file filtered using mean shift (kernel: 3D, 3 > max distance X & Y: 3 > noise spread: 1.0), unsharp (unit: 8 > kernel: 3D, 3 > standard deviation: 1.0 > unsharp factor: 1.0), and histogram balancing (reference slide #170).

*1.5_Cell_1_Stereocilia_Segmentation*^32^ is a multipage TIFF file illustrating stereocilia segmentation results. Same as ‘*Towers.am’* file used for *MATLAB* volume analysis of this cell, but converted to TIFF to allow for its online preview. The file contains 348 images, as all the planes that contained no segmentation data were removed.

*1.6_Cell_1_GoldParticle_Segmentation*^33^ is a multipage TIFF file illustrating the gold bead segmentation results. Same as ‘*GoldParticles.am’* file used for *MATLAB* volume analysis of this cell, but converted to TIFF to allow for its online preview. The file contains 348 images, as all the planes that contained no segmentation data were removed.

*1.7_Cell_1_Surface_Rendering*^34^ file is a compressed (zipped) folder containing all files required to render the surface of the bundle. Also included is the *‘ProjectLog’* file produced by Slice and View acquisition software.

*1.8_Cell_1_MATLAB_Volume_Analysis_Results*^35^ file is a compressed (zipped) folder containing all files required to execute the *MATLAB* volume analysis. The folder also includes the resulting output files and figures.

### Dataset 2

Includes the following files, all related to Cell#2 of the current study. The numbering order of the files reflects the order of operations performed during the analysis. Group link: http://cellimagelibrary.org/groups/50688.

*2.1_Cell_2_Raw_Image_Stack*^36^ is a multipage TIFF file consisting of 311 individual EM sections acquired at 10 nm milling step, resulting in 1.95 × 1.95 × 10 nm^3^ voxel size.

*2.2_Cell_2_Stack_Cropped*^37^ is a multipage TIFF file containing only the relevant volume of Cell#2, cropped to reduce the file size.

*2.3_Cell_2_Stack_Aligned*^38^ is a multipage TIFF file containing aligned images of Cell#2.

*2.4_Cell_2_Stack_Filtered*^39^ is a multipage TIFF file filtered using histogram balancing (set to reference image #137) and unsharp filter (kernel: 3D, 5 > standard deviation: 1.0 > unsharp factor: 6.0).

*2.5_Cell_2_Stereocilia_Segmentation*^40^ is a multipage TIFF file illustrating stereocilia segmentation results. Same as ‘*Towers.am’* file used for *MATLAB* volume analysis of this cell, but converted to TIFF to allow for its online preview.

*2.6_Cell_2_GoldParticle_Segmentation*^41^ is a multipage TIFF file illustrating the gold bead segmentation results. Same as ‘*GoldParticles.am’* file used for *MATLAB* volume analysis of this cell, but converted to TIFF to allow for its online preview.

*2.7_Cell_2_Surface_Rendering*^42^ file is a compressed (zipped) folder containing all files required to render the surface of the bundle. Also included is the *‘ProjectLog’* file produced by Slice and View acquisition software.

*2.8_Cell_2_MATLAB_Volume_Analysis_Results*^43^ file is a compressed (zipped) folder containing all files required to execute the *MATLAB* volume analysis. The folder also includes the resulting output files and figures.

### Dataset 3

Includes the following files, all related to Cell#3 of the current study. The numbering order of the files reflects the order of operations performed during the analysis. Group link: http://cellimagelibrary.org/groups/50697.

*3.1_Cell_3_Raw_Image_Stack*^44^ is a multipage TIFF file consisting of 208 individual EM sections acquired at 15 nm milling step, resulting in 2.6 × 2.6 × 15 nm^3^ voxel size. *Note:* some stereocilia are missing their tips and although segmented, were excluded from the *MATLAB* analysis.

*3.2_Cell_3_Stack_Cropped_Aligned*^45^ is a multipage TIFF file containing aligned images of Cell#3. Contains only the relevant volume of Cell#3, and was cropped to reduce the file size.

*3.3_Cell_3_Stack_Filtered*^46^ is a multipage TIFF file filtered with mean shift filter (kernel: 3D, 3 > max distance X & Y: 3 > noise spread: 1.0), unsharp filter (unit: 8 > kernel: 3D, 3 > standard deviation: 1.0 > unsharp factor: 1.0) and histogram balancing filter (set to reference image #170).

*3.4_Cell_3_Stereocilia_Segmentation*^47^ is a multipage TIFF file illustrating stereocilia segmentation results. Same as ‘*Towers.am’* file used for *MATLAB* volume analysis of this cell, but converted to TIFF to allow for its online preview.

*3.5_Cell_3_GoldParticle_Segmentation*^48^ is a multipage TIFF file illustrating the gold bead segmentation results. Same as ‘*GoldParticles.am’* file used for *MATLAB* volume analysis of this cell, but converted to TIFF to allow for its online preview.

*3.6_Cell_3_Surface_Rendering*^49^ file is a compressed (zipped) folder containing all files required to render the surface of the bundle. Also included is the *‘ProjectLog’* file produced by Slice and View acquisition software.

*3.7_Cell_3_MATLAB_Volume_Analysis_Results*^50^ file is a compressed (zipped) folder containing all files required to execute the *MATLAB* volume analysis. Folder also includes the resulting output files and figures.

### Dataset 4

Includes the following files, all related to Cell #4 of the current study. The numbering order of the files reflects the order of operations performed during the analysis. *Note:* The data acquisition session was interrupted after frame #224, and later resumed as a separate imaging session. This resulted in two stack files that were subsequently concatenated into a single multipage TIFF file. Group link: http://cellimagelibrary.org/groups/50704.

*4.1_Cell_4_Raw_Image_Stack_Part1*^51^ is a multipage TIFF file consisting of 224 individual EM sections acquired at 15 nm milling step, resulting in 1.95 × 1.95 × 15 nm^3^ voxel size.

*4.2_Cell_4_Raw_Image_Stack_Part2*^52^ is a multipage TIFF file consisting of an additional 144 EM sections acquired completing Cell#4. Images were acquired at 15 nm milling step, resulting in 1.95 × 1.95 × 15 nm^3^ voxel size.

*4.3_Cell_4_Raw_Image_Stack*^53^ is a multipage TIFF file consisting of 368 individual EM sections representing the entire volume of Cell#4.

*4.4_Cell_4_Stack_Cropped_Aligned*^54^ is a multipage TIFF file containing aligned images of Cell#4, which were cropped to contain only the relevant volume, and reduce the file size.

*4.5_Cell_4_Stack_Filtered* is a multipage TIFF file filtered with slope map filter (unit: 8 > scale: 10), unsharp filter (unit: 8 > kernel: 3D, 5 > standard deviation: 1.0 > unsharp factor: 0.0). The file contains 368 images.

*4.6_Cell_4_Stereocilia_Segmentation*^56^ is a multipage TIFF file illustrating stereocilia segmentation results. Same as ‘*Towers.am’* file used for *MATLAB* volume analysis of this cell, but converted to TIFF to allow for its online preview. The file contains 362 images, as all the planes that contained no segmentation data were removed.

*4.7_Cell_4_GoldParticle_Segmentation*^57^ is a multipage TIFF file illustrating the gold bead segmentation results. Same as ‘*GoldParticles.am’* file used for *MATLAB* volume analysis of this cell, but converted to TIFF to allow for its online preview. The file contains 362 images, as all the planes that contained no segmentation data were removed.

*4.8_Cell_4_Surface_Rendering*^58^ file is a compressed (zipped) folder containing all files required to render the surface of the bundle. Also included is the *‘ProjectLog’* file produced by Slice and View acquisition software.

*4.9_Cell_4_MATLAB_Volume_Analysis_Results*^59^ file is a compressed (zipped) folder containing all files required to execute the *MATLAB* volume analysis. The folder also includes the resulting output files and figures.

### Dataset 5

Includes the following files, all related to Cell #5 of the current study. The numbering order of the files reflects the order of operations performed during the analysis. Group link: http://cellimagelibrary.org/groups/50713.

*5.1_Cell_5_Raw_Image_Stack*^60^ is a multipage TIFF file consisting of 233 individual EM sections acquired at 20 nm milling step, resulting in 2.75 × 2.75 × 20 nm^3^ voxel size.

*5.2_Cell_5_Stack_Cropped_Aligned*^61^ is a multipage TIFF file containing aligned images of Cell#5, which were cropped to contain only the relevant volume, and reduce the file size.

*5.3_Cell_5_Stack_Filtered*^62^ is a multipage TIFF file filtered with unsharp filter (unit: 8 > kernel: 3D, 5 > standard deviation: 1.0 > unsharp factor: 1.0).

*5.4_Cell_5_Stereocilia_Segmentation*^63^ is a multipage TIFF file illustrating stereocilia segmentation results. Same as ‘*Towers.am’* file used for *MATLAB* volume analysis of this cell, but converted to TIFF to allow for its online preview.

*5.5_Cell_5_GoldParticle_Segmentation*^64^ is a multipage TIFF file illustrating the gold bead segmentation results. Same as ‘*GoldParticles.am’* file used for *MATLAB* volume analysis of this cell, but converted to TIFF to allow for its online preview.

*5.6_Cell_5_Surface_Rendering*^65^ file is a compressed (zipped) folder containing all files required to render the surface of the bundle. Also included is the *‘ProjectLog’* file produced by Slice and View acquisition software.

*5.7_Cell_5_MATLAB_Volume_Analysis_Results*^66^ file is a compressed (zipped) folder containing all files required to execute the *MATLAB* volume analysis. The folder also includes the resulting output files and figures.

### Dataset 6

Includes the following files, all related to Cell #6 of the current study. The numbering order of the files reflects the order of operations performed during the analysis. Group link: http://cellimagelibrary.org/groups/50720.

*1.1_Cell_1_and_Cell6_Raw_Image_Stack*^28^ (a part of *Dataset 1*) is a multipage TIFF file consisting of 388 individual EM sections acquired at 15 nm milling step, resulting in 2.43 × 2.43 × 15 nm^3^ voxel size. This file also contains the raw data related to Cell#1.

*Note:* The data acquisition session was interrupted after frame #161 to adjust the imaging area, resulting in a large shift on subsequent frame #162.

*6.2_Cell_6_Stack_Aligned_Cropped*^67^ is a multipage TIFF file containing aligned, then cropped images of Cell#6, which were cropped to contain only the relevant volume, and reduce the file size. No filters were used to analyze this dataset. File contains 324 images, as all the planes that contained no relevant data were removed.

*6.3_Cell_6_Stereocilia_Segmentation*^68^ is a multipage TIFF file illustrating stereocilia segmentation results. Same as ‘*Towers.am’* file used for *MATLAB* volume analysis of this cell, but converted to TIFF to allow for its online preview. File contains 324 images, as all the planes that contained no segmentation data were removed.

*6.4_Cell_6_GoldParticle_Segmentation*^69^ is a multipage TIFF file illustrating the gold bead segmentation results. Same as ‘*GoldParticles.am’* file used for *MATLAB* volume analysis of this cell, but converted to TIFF to allow for its online preview. File contains 324 images, as all the planes that contained no segmentation data were removed.

*6.5_Cell_6_Surface_Rendering*^70^ file is a compressed (zipped) folder containing all files required to render the surface of the bundle. Also included is the *‘ProjectLog’* file produced by Slice and View acquisition software.

*6.6_Cell_6_MATLAB_Volume_Analysis_Results*^71^ file is a compressed (zipped) folder containing all files required to execute the *MATLAB* volume analysis. Folder also includes the resulting output files and figures. Also included is the “*All Cells combined gold bead quantification results*” table with all gold bead quantification results across all six cells used in this study.

### Dataset 7

Technical Validation dataset. Group link: http://cellimagelibrary.org/groups/50725.

*7.1_Test_Cell_Stereocilia_Segmentation*^72^ is a multipage TIFF file illustrating stereocilia segmentation results of Cell#3. Same as ‘*Towers.am’* file used for *MATLAB* volume analysis of this cell, but converted to TIFF to allow for online preview.

*7.2_Test_Cell_GoldParticle_Segmentation*^73^ is a multipage TIFF file illustrating the manually placed gold beads in a pattern as described in the *Technical Validation* section. Same as ‘*GoldParticles.am’* file used for *MATLAB* volume analysis of this cell, but converted to TIFF to allow for online preview.

*7.3_Test_Cell_MATLAB_Volume_Analysis_Results*^74^ file is a compressed (zipped) folder containing all files required to execute the *MATLAB* volume analysis. The folder also includes the resulting output files and figures.

### Dataset 8

*MATLAB* analysis algorithm^75^. *MATLAB script* is a compressed (zipped) folder containing the *MATLAB* script files required to perform the abovementioned analysis (10.7295/w9cil50728).

## Technical Validation

**Fig. 8:**
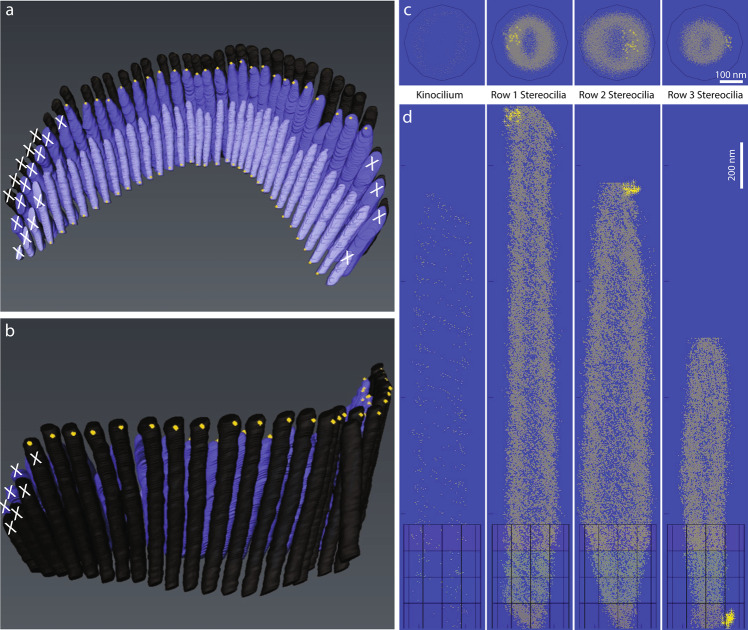
*MATLAB* script technical validation. (**a,b**) An artificial dataset with manually placed gold beads (yellow dots) was used to access the accuracy of the MATLAB analysis workflow, experimentally validating its performance. All stereocilia excluded from the analysis were labeled with white *‘X’* marks. (**c,d**) A 3D cumulative distribution map of manually placed gold beads generated by the *MATLAB* script. Top and side views (*c* and *d*, respectively) are shown for the kinocilium (left) and three rows of stereocilia. Yellow asterisks represent gold beads; gray dots represent the convex hull points of stereociliary surface. Illustrated gold beads are tightly clustered within the locations where they were intentionally placed by the authors. The choice of gold bead placement location was unique for each row of stereocilia to control for potential errors of data processing such as stereocilia orientation, azimuth correction, or placement within the rows during *MATLAB* analysis. No beads were placed on the surface of the kinocilium.

A manually generated test **Dataset 7**^72–74^ was used to evaluate the performance of the *MATLAB* algorithm, testing it for any potential errors. One of the existing stereocilia segmentation files was used, in which all gold beads were removed. Using this data set, we manually placed a single gold bead per stereocilium, but varied its location among the rows of stereocilia (Fig. 8a,b). For the tallest row, the bead was placed at the tip on the surface opposite from the middle row stereocilia. For the middle row, the bead was placed on the surface away from the tallest row cilia; and for the short row stereocilia it was placed at the lower end near the cuticular plate, on the surface opposite from the middle row stereocilia. No beads were placed on the surface of the kinocilium. This provided a control for script’s performance in the following tasks:

- every tower (stereocilium) is properly oriented in space (for example, was not positioned upside down when its principal directions were computed);
- every tower (stereocilium) is properly identified within the rows, as assigned by the user in the ‘*LinkTable.csv*’ file. That is evident, as each row in the test set has its unique, row-specific placement of the gold bead. In the event of any stereocilia mistakenly assigned to the wrong row, any misplaced gold beads would be evident in the resulting gold bead distribution map (Fig. 8c,d);
- all towers were properly oriented during the azimuth transformation step: although without a high precision, each gold bead was manually placed as outlined above, and all of them were tightly clustered within the proper segment of stereocilia following the azimuth transform step.
- each gold bead is assigned to the surface of the nearest stereocilium. In the event of any gold bead mistakenly assigned to the wrong stereocilium, such misplaced gold beads would appear misplaced in the resulting gold bead distribution map (Fig. 8c,d**)**.

As seen from the gold bead distribution map on Fig. 8c,d, all stereocilia and gold beads were identified and analyzed as expected, validating the performance of the *MATLAB* algorithm in performing all abovementioned tasks.

## Data Availability

This study was carried out using the following software packages: Dragonfly 2017 by Object Research Systems, free for non-commercial users, and available for download at https://www.theobjects.com/dragonfly/index.html. Amira 6.2.0 by Thermo Fisher Scientific. *MATLAB* 2017a by MathWorks. The *MATLAB* script is deposited with *The Cell Image Library* (10.7295/w9cil50728), available for download as **Dataset 8**^75^ of this submission.
